# Exosome engineering for efficient intracellular delivery of soluble proteins using optically reversible protein–protein interaction module

**DOI:** 10.1038/ncomms12277

**Published:** 2016-07-22

**Authors:** Nambin Yim, Seung-Wook Ryu, Kyungsun Choi, Kwang Ryeol Lee, Seunghee Lee, Hojun Choi, Jeongjin Kim, Mohammed R. Shaker, Woong Sun, Ji-Ho Park, Daesoo Kim, Won Do Heo, Chulhee Choi

**Affiliations:** 1 Department of Bio and Brain Engineering, KAIST, Daejeon 34141, Korea; 2 Cellex Life Sciences Inc., Daejeon 34141, Korea; 3 Department of Biological Sciences, KAIST, Daejeon 34141, Korea; 4 Department of Anatomy, Brain Korea 21 Program, Korea University College of Medicine, Seoul 02841, Korea; 5 Cancer Metastasis Control Center, KAIST Institute for the Biocentury, KAIST, Daejeon 34141, Korea; 6 Center for Cognition and Sociality, Institute for Basic Science (IBS), Daejeon 34047, Korea

## Abstract

Nanoparticle-mediated delivery of functional macromolecules is a promising method for treating a variety of human diseases. Among nanoparticles, cell-derived exosomes have recently been highlighted as a new therapeutic strategy for the *in vivo* delivery of nucleotides and chemical drugs. Here we describe a new tool for intracellular delivery of target proteins, named ‘exosomes for protein loading via optically reversible protein–protein interactions' (EXPLORs). By integrating a reversible protein–protein interaction module controlled by blue light with the endogenous process of exosome biogenesis, we are able to successfully load cargo proteins into newly generated exosomes. Treatment with protein-loaded EXPLORs is shown to significantly increase intracellular levels of cargo proteins and their function in recipient cells *in vitro* and *in vivo*. These results clearly indicate the potential of EXPLORs as a mechanism for the efficient intracellular transfer of protein-based therapeutics into recipient cells and tissues.

## Abstract (web summary)

Exosomes have been identified as promising vehicles for the *in vivo* delivery of therapeutic molecules. Here the authors design a system to load protein cargos into exosomes during their biogenesis using optogenetic control of protein-protein interactions between the cargo and an exosome-localized partner.

## Graphical abstract

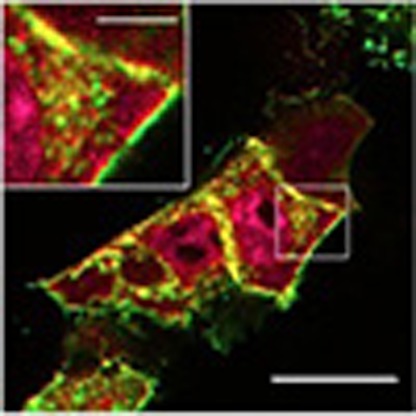

Despite the long list of therapeutic proteins available for treating various human diseases, the vast majority of clinically available protein-based drugs, such as cytokines, hormones and monoclonal antibodies, have been limited to extracellular mechanisms of action. Intracellular proteins have been identified for their potential as biopharmaceutical drugs; however, many of the challenges associated with intracellular protein delivery have yet to be solved^1^. Protein transduction methods have been proposed to deliver recombinant proteins into target cells *in vitro* and *in vivo*^2^,^3^,^4^. Although the techniques are promising, exposure of the protein surface in solution and the low refolding rate of recombinant proteins in target cells remain important obstacles for the intracellular delivery of therapeutic proteins^5^. Several lipid nanoparticle-mediated protein delivery methods have been postulated, as has protein encapsulation for protecting the proteins^6^,^7^. However, the absence of a separation mechanism between cargo proteins and lipid nanoparticles not only limits the efficiency of cytosolic delivery, preparation of these particles often involves complicated protein purification steps.

To address these limitations, we developed an optogenetically engineered exosome system EXPLORs (exosomes for protein loading via optically reversible protein–protein interactions) that can deliver soluble proteins into the cytosol via controlled, reversible protein–protein interactions (PPIs). Exosomes are natural cell-derived extracellular vesicles that originate from internal endocytic compartments and multi-vesicular bodies and participate in intercellular communication^8^. Recent studies have sought to use exosomes as a new method for the *in vivo* delivery of siRNA or miRNA to specific target tissues by systemic injection^9^,^10^,^11^. These methods were based on the passive loading of siRNAs or miRNAs into isolated exosomes by electrophoresis, a method poorly suited for loading of macromolecular proteins. Here we propose a novel protein-loading method in which cargo proteins can be actively loaded into exosomes through endogenous biogenesis processes, allowing for efficient delivery into the cytosol of target cells through controllable, reversible detachment from the exosomes.

To achieve the controllable, reversible loading and delivery of target proteins into the exosome, we selected a photoreceptor cryptochrome 2 (CRY2), and CRY-interacting basic-helix-loop-helix 1 (CIB1) protein module, originally identified in *Arabidopsis thaliana*, which regulates floral initiation via blue light-dependent phosphorylation^12^,^13^,^14^. We induced a transient docking of CRY2-conjugated cargo proteins to the exosomes by introducing CIBN (a truncated version of CIB1)^14^ conjugated with an exosome-associated tetraspanin protein CD9 and blue light illumination (Fig. 1a,b). Once the cargo proteins are introduced into the exosomes via the process of endogenous biogenesis, they can be detached from CD9-conjugated CIBN by removal of the illumination source, resulting in their release into the intraluminal space of the exosomes and enabling efficient delivery to the cytosolic compartment of target cells.

## Results

### Light-inducible PPI module for producing EXPLORs

**Figure 1:**
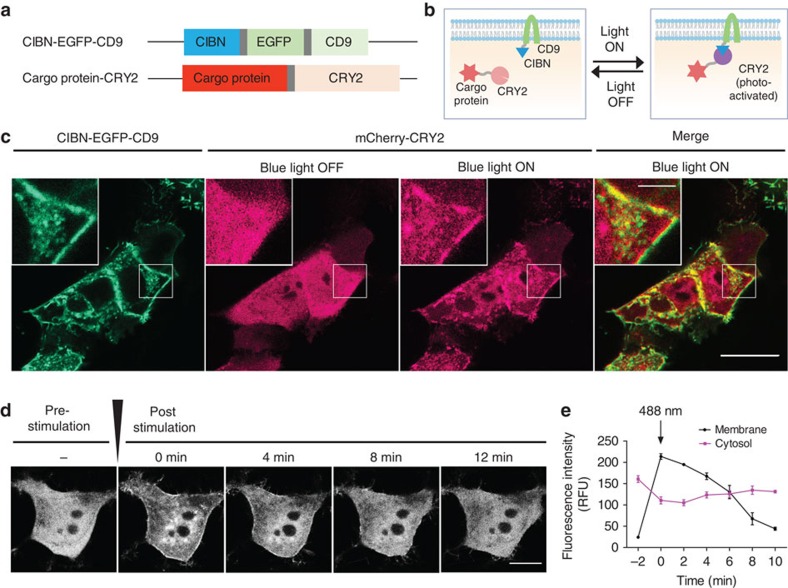
Generation of engineered EXPLOR. (**a**) Schematics of DNA constructs used for the production of EXPLOR. (**b**) Schematic showing fusion proteins and their proposed action. (**c**) HEK293T cells were transiently transfected with *CIBN-EGFP-CD9* and *mCherry-CRY2* expression vectors. The mCherry fluorescence was imaged before and after 488-nm laser stimulation (15 s in duration, 350 μW cm^−2^). Scale bars, 20 μm (5 μm for inset images). A representative result from at least 10 experiments. (**d**) HEK293T cells transiently transfected with *CIBN-EGFP-CD9* and *mCherry-CRY2* were imaged for time-lapse imaging of mCherry fluorescence for varying time periods (0–12 min) after a stimulation (black arrow) of 488-nm light (15 s in duration, 350 μW cm^−2^). Scale bars, 5 μm. A representative result of at least 10 experiments. (**e**) Quantification of mCherry fluorescence in the cytoplasm and at the plasma membrane. Data are presented as the mean±s.e.m. (*n*=3).

We generated HEK293T cells that can produce EXPLORs loaded with mCherry (mCherry:EXPLOR) as a target protein by introducing vectors for two fusion proteins: CIBN-conjugated enhanced green fluorescent protein (EGFP)-tagged CD9 (CIBN-EGFP-CD9) and mCherry-tagged CRY2 (mCherry-CRY2). A single pulse irradiation of a 488-nm laser induced rapid movement of mCherry-CRY2 from the cytosolic compartment to the plasma membrane and intracellular compartments where the CIBN-EGFP-CD9 proteins were co-localized (Fig. 1c and Supplementary Fig. 1a). Similar results were obtained by the introduction of another photoreceptor module GIGANTEA, and LOV (light-, oxygen- and voltage-sensitive domain) proteins^15^ and other exosome-enriched tetraspanin proteins, such as CD63, CD81 and CD82 (Supplementary Fig. 1b,c). We could also observe light-induced co-localization of mCherry-CRY2 and CIBN-EGFP-CD9 by a cryo-immunogold electron microscopy analysis (Supplementary Fig. 2). Introduction of a mutant CRY2, mutated at an amino acid important in the photolyase function (D387A)^16^, abrogated such light-induced co-localization of CIBN-EGFP-CD9 and mCherry-CRY2 (Supplementary Fig. 3). On termination of light stimulation, the association between CIBN-EGFP-CD9 and mCherry-CRY2 dissociated gradually within 10 min, as described previously^14^ (Fig. 1d,e).

### Light-dependent loading of proteins in EXPLORs

**Figure 2:**
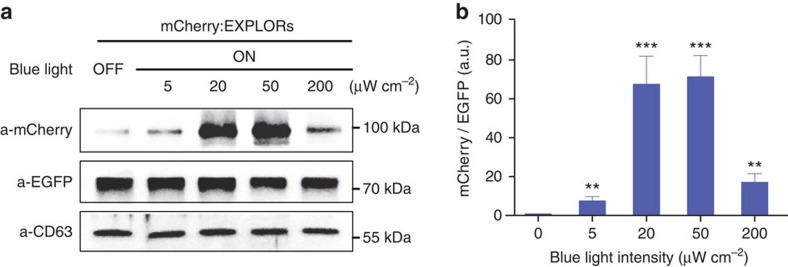
Light-dependent loading of target proteins in EXPLORs. (**a**) Cells transiently transfected with *CIBN-EGFP-CD9* and *mCherry-CRY2* expression vectors were maintained under blue light illumination of varying powers for 48 h. Cell-derived exosomes were subject to immunoblot analysis using antibodies against mCherry, EGFP and CD63, an exosome marker. A representative result from three independent experiments. (**b**) The graph presents densitometry analysis for normalized amount of mCherry-CRY2 protein over CIBN-EGFP-CD9 protein from three independent experiments. Data are presented as the mean±s.e.m. (*n*=3), and Tukey's *post hoc* test was applied to significant group effects (**P<0.01, ***P<0.001) identified by analysis of variance.

For production of mCherry:EXPLORs, we exposed transiently transfected HEK293T cells with continuous blue light illumination by installing a 460-nm light-emitting diode (LED; maximum power; 380 μW cm^−2^) in a CO_2_ incubator. After 48-h incubation with blue light illumination, EXPLORs were isolated from the supernatants by several different methods and confirmed for the hydrodynamic diameter by dynamic light scattering (Supplementary Fig. 4 and Supplementary Table 1). Immunoblotting results demonstrated that EXPLORs derived from optogenetically engineered HEK293T cells grown under blue light illumination contained significantly higher amounts of mCherry-CRY2 protein (84 kDa) than those obtained from cells grown in the dark (Fig. 2a and Supplementary Fig. 5). In particular, the highest efficiency in protein loading was seen in the power range from 20 to 50 μW cm^−2^ (Fig. 2b). The threshold power of the light-induced PPI was measured at ∼15 μW cm^−2^ by quantified laser stimulation (Supplementary Fig. 6) and at ∼20 μW cm^−2^ by the LED light (Supplementary Fig. 7). Continuous irradiation with blue light for up to 48 h did not induce significant cell death (Supplementary Fig. 8), and we further confirmed that EXPLORs did not contain detectable DNA fragments typical of apoptotic bodies (Supplementary Fig. 9a). Because genetically modified cells may release exosomes packed with not only the encoded protein but also genetic material (pDNA and mRNA) encoding this protein^17^, we further examined EXPLORs containing undetectable amounts of plasmid DNA (data not shown) and mCherry-CRY2 mRNA (Supplementary Fig. 9b). In addition, we tested the loading efficiency of mCherry proteins in EXPLORs in response to cycles of blue light illumination and darkness. Using a variety of cycle combinations, we identified a cycle consisting of 1 min of blue light illumination followed by 1 min of darkness as the optimal condition to produce mCherry:EXPLORs (Supplementary Fig. 10).

**Figure 3:**
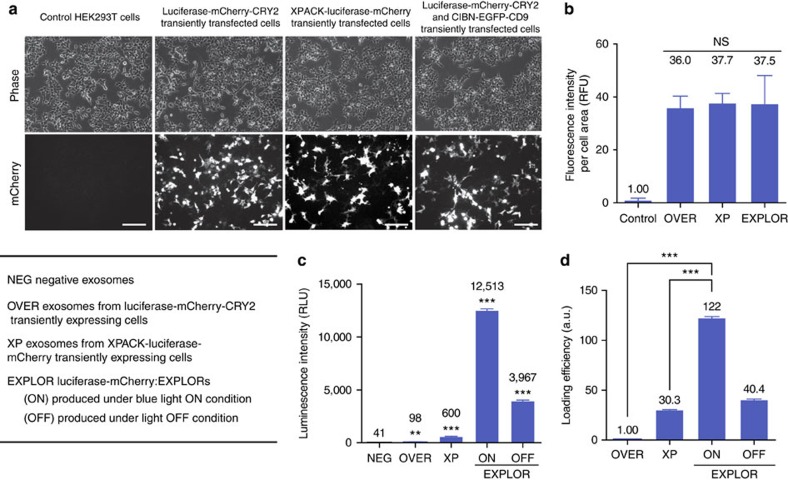
Comparison of the exosome-loading capacity of target proteins between various protein-loading methods. (**a**) HEK293T cells were transiently transfected with *luciferase-mCherry-CRY2* expression vector alone, *XPack-luciferase-mCherry* expression vector or co-transfected with *CIBN-EGFP-CD9* and *luciferase-mCherry-CRY2* expression vectors. After 24 h, cells were imaged by fluorescence microscopy for the expression profile of mCherry fusion proteins. A representative result from five independent experiments. Scale bars, 20 μm. (**b**) Quantification of mCherry fluorescence. Data are presented as the mean±s.e.m. (*n*=5), and Tukey's *post hoc* test was applied to significant group effects identified by analysis of variance (ANOVA). Control: untransfected HEK293T cells; OVER: cells transiently transfected with a *luciferase-mCherry-CRY2* vector; XP: cells transfected with an *XPACK-luciferase-mCherry* vector; and EXPLOR: cells transfected with both *luciferase-mCherry-CRY2* and *CIBN-EGFP-CD9* vectors. (**c**) Cells transiently transfected with various vectors were maintained for 48 h. In the case of EXPLOR-producing cells, cells were maintained in the absence (OFF) or presence (ON) of blue light illumination; 5 × 10^8^ particles of the isolated exosomes were analysed for luciferase activity. Data are presented as the mean±s.e.m. (*n*=3), and Tukey's *post hoc* test was applied to significant group effects (**P<0.01, ***P<0.001) identified by ANOVA. (**d**) Loading efficiency of various protein-loaded exosomes was calculated by dividing the number of luciferase molecules in exosomes with the number of luciferase molecules in the exosome-producing cells. Numbers of luciferase molecules were estimated from a standard curve using recombinant luciferase. Data are presented as the mean±s.e.m. (*n*=3), and Tukey's *post hoc* test was applied to significant group effects (***P<0.001) identified by ANOVA. NS, not significant.

We next examined the loading capacity of EXPLOR technology by producing firefly luciferase-containing EXPLORs. For the production of luciferase:EXPLOR, we cloned vectors for firefly luciferase-conjugated mCherry-CRY2 and confirmed light-induced PPI with CIBN-EGFP-CD9 by blue light stimulation with confocal microscopy (Supplementary Fig. 11). First, we compared luciferase:EXPLORs with a commercialized method for exosome protein loading, XPACK (System Biosciences, Mountain View, CA, USA), which is based on the principle that highly oligomeric, cytoplasmic proteins can be targeted to exosomes by plasma membrane anchors^18^,^19^. We also included exosomes produced from cells transfected with the luciferase-conjugated mCherry-CRY2 vector, but not with the CIBN-EGFP-CD9 expression plasmid, to exclude the possibility of nonspecific loading of overexpressed cargo proteins into exosomes. Transfection efficiency and expression profile of luciferase-mCherry fusion proteins were comparable between the different methods (Fig. 3a,b and Supplementary Fig. 12). For the quantitative analysis of cargo protein loading into the exosomes, we measured luciferase activity using the same numbers of isolated exosomes (Fig. 3c,d). The results showed that the loading capacity of EXPLORs was significantly higher than those of the overexpression model of luciferase-mCherry-CRY2 and a commercialized model for exosome protein targeting. In our experiment, it was estimated that, on average, 1.4 molecules of luciferase proteins were included in each exosome obtained by EXPLOR technology (Supplementary Fig. 13). We also tested another method: *ex vitro* protein loading in naive exosomes loaded with recombinant luciferases. In total, 50 μg of recombinant luciferases (61 kDa) dissolved in phosphate-buffered saline (PBS) was added to 2 × 10^10^ exosomes derived from HEK293T cells and loaded into exosomes *ex vitro* with an extrusion method^20^. Only 4.4 pg of recombinant protein was finally included in the exosomes; thus, the loading capacity of the *ex vitro* protein-loading method was estimated to be 40-fold lower than that of the EXPLOR technology (Supplementary Fig. 14).

### Administration of EXPLORs *in vitro* and *in vivo*

**Figure 4:**
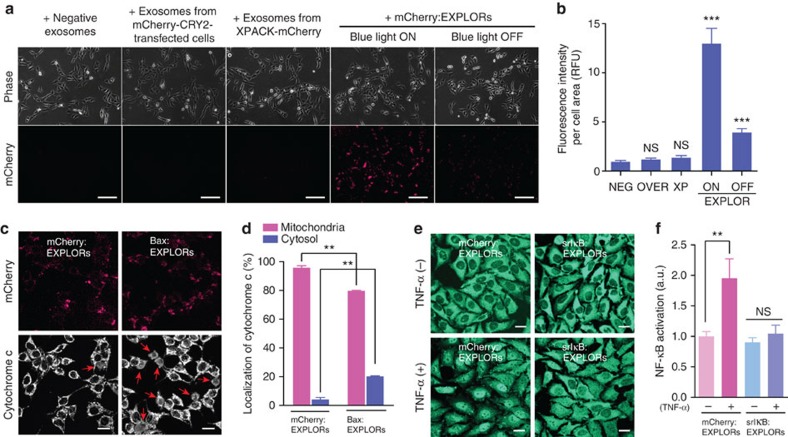
EXPLOR-mediated intracellular delivery of cargo proteins. (**a**,**b**) HeLa cells were incubated in the absence or presence of 5 × 10^9^ particles of various isolated exosomes for 24 h and imaged by fluorescence microscopy. The fluorescence intensities of mCherry were quantified by two imaging processing tools, ImageJ and Cellprofiler. Data are presented as the mean±s.e.m. (*n*=15), and Tukey's *post hoc* test was applied to significant group effects (***P<0.001) identified by analysis of variance (ANOVA). Scale bars, 100 μm. (**c**,**d**) HeLa cells were incubated in the absence or presence of 0.1 mg ml^−1^ mCherry:EXPLORs or Bax-mCherry:EXPLORs for 12 h, and fixed with 4% paraformaldehyde. Then, cytochrome *c* was stained with an antibody conjugated with Alexa Fluor 647 and imaged by confocal microscopy. The ratios of cytochrome *c* localization were analysed by cell counting. Data are presented as the mean±s.e.m. (*n*=3), and Tukey's *post hoc* test was applied to significant group effects (**P<0.01) identified by ANOVA. Scale bars, 20 μm. (**e**) HeLa cells were incubated in the absence or presence of 0.1 mg ml^−1^ mCherry:EXPLORs or srIκB:EXPLORs for 12 h, treated with 10 ng ml^−1^ tumour necrosis factor- α (TNF-α) for an additional 30 min, and fixed with 4% paraformaldehyde. NF-κB p65 was stained with an antibody conjugated with Alexa Fluor 488 and imaged by confocal microscopy. (**f**) The nuclear extracts of cells were assayed for the DNA-binding activity of p65/c-Rel (NF-κB). Data are presented as the mean±s.e.m. (*n*=3), and Tukey's *post hoc* test was applied to significant group effects (**P<0.01) identified by ANOVA. Scale bars, 20 μm. NS, not significant.

HeLa cells were incubated in the absence or presence of mCherry-loaded exosomes for 24 h, and tested for analysis with EXPLOR-mediated protein delivery to recipient cells (Fig. 4a). Consistent with previous results, EXPLOR-treated cells showed significantly higher fluorescence signals (Fig. 4b). Cryo-immunogold electron microscopy analysis (Supplementary Fig. 15a) and confocal microscopy (Supplementary Fig. 15b) revealed that mCherry proteins were clearly transferred into the cytosolic region of cells after incubation with mCherry:EXPLORs derived under the ‘light ON' condition. Immunoblot analysis demonstrated significant increases in the amounts of intracellular mCherry proteins in recipient cells after treatment with mCherry:EXPLORs in both a time- and dose-dependent manner (Supplementary Fig. 15c,d).

To further confirm whether EXPLOR technology can be useful for the delivery of functional proteins, we cloned vectors for mCherry-CRY2 proteins fused with Bax and super-repressor IκB (srIκB) proteins (Supplementary Fig. 16). In the normal condition, Bax proteins are mainly distributed in the cytosol; however, on initiation of the apoptotic process, they undergo a conformation change and move to the mitochondrial membrane, leading to the release of cytochrome *c* that subsequently triggers caspase-dependent apoptosis^21^,^22^,^23^,^24^. As expected, incubation with Bax-mCherry:EXPLORs induced a rapid release of cytochrome *c* from the mitochondria in HeLa cells (Fig. 4c,d). Similarly, the endogenous nuclear factor-κB (NF-κB) inhibitor IκB proteins bind to the NF-κB complex in the cytoplasm; however, on relevant stimulation, IκB is phosphorylated by IκB kinases and degraded by the ubiquitin–proteasome pathway. Then, the NF-κB complexes translocate to the nuclei and induce the expression of survival- and inflammation-related genes^25^. Super-repressor IκB, a S32A and S36A mutant form of IκB, is not phosphorylated by IκB kinases, so that srIκB suppresses the translocation of NF-κB complex even in the presence of pro-inflammatory stimulation^26^. As expected, treatment with srIκB-mCherry:EXPLORs significantly reduced tumour necrosis factor-α-induced translocation of the p65 subunit of NF-κB in HeLa cells (Fig. 4e,f).

**Figure 5:**
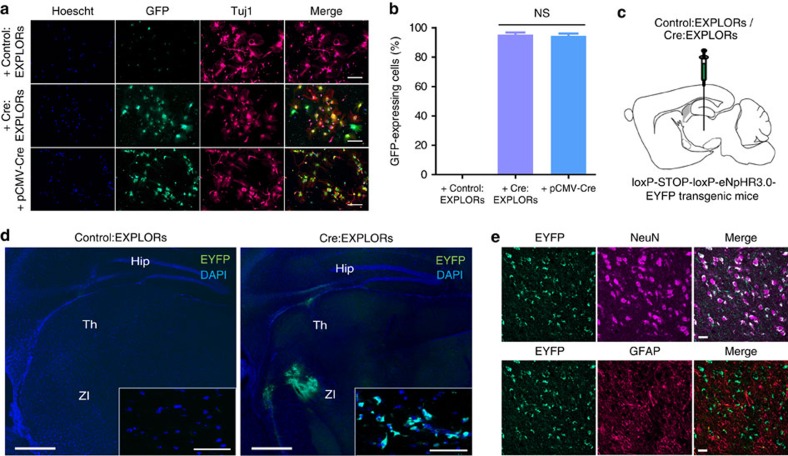
EXPLOR-mediated delivery of Cre recombinase *in vitro* and *in vivo*. (**a**,**b**) Differentiated neurosphere-derived cells were incubated in the absence or presence of 2 × 10^10^ particles per ml of Cre:EXPLORs (0.16 mg ml^−1^) or transfected with *pCMV-Cre* vector for 72 h. Cells were fixed with 4% paraformaldehyde and immune-stained with antibodies against a neuron-specific class III beta-tubulin marker, Tuj1, GFP and Hoechst 33342. The ratios of EGFP-expressing cells were analysed by cell counting. Data are presented as the mean±s.e.m. (*n*=10 fields), and Tukey's *post hoc* test was applied to significant group effects identified by ANOVA. Scale bars, 100 μm. (**c**) An experimental scheme for the administration of Cre:EXPLORs in *loxp-stop-loxp-eNpHR3.0-EYFP* transgenic mice. In total, 50 μl of Cre:EXPLORs (10 mg ml^−1^) were administered to *pCAG-loxP-STOP-loxP-eNpHR3.0-EYFP* transgenic mice by ventrolateral injection. (**d**) Brain slices of EXPLOR-injected transgenic mice were fixed with 4% formaldehyde and imaged by fluorescence microscopy. Green fluorescence indicates eNpHR3.0-EYFP protein expression and blue fluorescence indicates cell nuclei. Inset images showed confocal microscopy images of the detailed cellular eNpHR3.0-EYFP expression in EXPLOR-administered mouse neurons of the zona incerta (ZI) region. Scale bars, 500 μm (50 μm for inset confocal images). Hip, hippocampus; Th, thalamus. A representative of two independent experiments. (**e**) Representative image of NeuN/GFAP immunohistochemistry of the brain. Pink, neuronal-specific nuclear protein (NeuN)-positive neurons; Red, glial fibrillary acidic protein (GFAP)-positive astrocyte cells. Objective lens, × 40. Scale bar, 20 μm. NS, not significant.

Finally, we validated the potential of EXPLOR-mediated intracellular protein delivery for *in vivo* application. To test the intracellular delivery of Cre recombinase containing EXPLORs (Cre:EXPLORs), we cloned a vector for Cre-conjugated mCherry-CRY2 (Cre-mCherry-CRY2) protein. Unlike mCherry-CRY2 protein, Cre-mCherry-CRY2 proteins were predominantly expressed in the nuclei, and blue light illumination induced limited translocation of these proteins to the plasma membrane and intracellular endosomal fractions (Supplementary Fig. 17a). To avoid unwanted nuclear localization and increase the loading efficiency of Cre-mCherry-CRY2 proteins, cells were maintained under blue light illumination immediately after transfection. As expected, continuous light illumination immediately after transfection markedly enhanced co-localization of Cre-mCherry-CRY2 proteins with CIBN-EGFP-CD9 (Supplementary Fig. 17b). The enzymatic activity of Cre recombinase was validated in the target cells previously transfected with a reporter plasmid containing a *loxP-STOP-loxP* sequence upstream of the *ZsGreen*-encoding gene. Treatment with Cre:EXPLORs clearly induced green fluorescence at levels comparable to that of transfection of *pCMV-Cre* vectors in various cells including rat embryonic primary neurons (Supplementary Fig. 18). Because the transient transfection of primary neurons is extremely inefficient, we next tested the effect of Cre:EXPLORs on neurosphere-derived differentiated neuronal cells isolated from transgenic mice having a *loxP-STOP-loxP-EGFP* gene. As expected, treatment with Cre:EXPLORs induced EGFP expression in over 95% of cells; while treatment with naive EXPLORs had no effect (Fig. 5a,b). Next, we injected Cre:EXPLORs into the ventrolateral part of the brain of *pCAG-loxP-STOP-loxP-eNpHR3.0-EYFP* (enhanced yellow fluorescence protein) transgenic mice (Fig. 5c). After 96 h, eNpHR3.0 EYFP proteins were expressed widely near the ventrolateral part in the brain, and especially were expressed highly in the zona incerta region (Fig. 5d). Immunohistochemical analyses demonstrated that the EYFP-positive cells were mainly neurons, indicating that Cre:EXPLORs worked functionally mainly in neurons *in vivo* (Fig. 5e and Supplementary Fig. 19).

## Discussion

**Figure 6:**
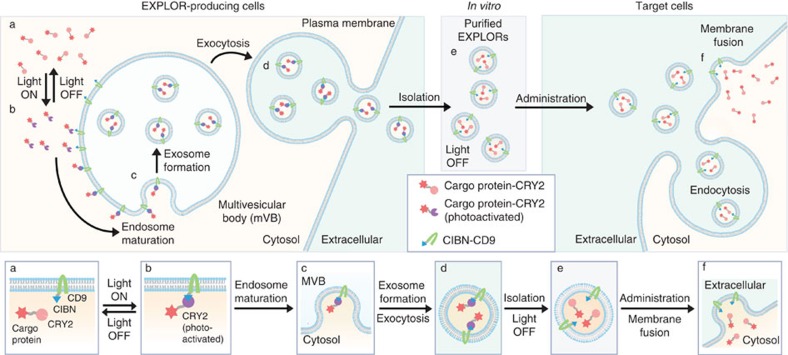
Schematic diagram of EXPLOR technology. In EXPLOR-producing donor cells, CRY2 protein was fused to a cargo protein, and CIBN was conjugated with a representative marker of exosomes, CD9 protein. Blue light illumination induces the reversible PPI between CIBN and CRY2 fusion proteins. With continuous blue light irradiation, the cargo proteins are guided to the inner surface of the cell membrane or the surface of early endosomes. Mature multi-vesicular bodies (MVBs) then readily secrete cargo protein-carrying exosomes (EXPLORs) from the cells by membrane fusion with the plasma membrane. After exocytosis, EXPLORs can be easily isolated and purified *in vitro*. Purified EXPLORs can be used for delivery of the cargo proteins into target cells via membrane fusion or endocytosis processes. Bottom grey boxes highlight the essential steps from EXPLORs biogenesis to target cell delivery.

Although protein transduction and lipid nanoparticle-mediated protein delivery methods have been proposed for direct protein delivery into target cells and tissues^6^,^7^,^27^, many obstacles remain before these methods can be successfully used *in vivo*, including low purification efficiency, failure to separate from nanoparticles in recipient cells and induction of immune responses against host immune cells. To address these limitations, we developed an exosome-based delivery system EXPLORs that can be readily produced using live cells as a factory via the endogenous biogenesis of extracellular vesicles (Fig. 6).

This novel method provides significant advantages for both research and clinical applications. First, EXPLORs can carry cargo proteins without isolation or purification of recombinant proteins. Because EXPLORs contain cell-produced cargo proteins in unbound soluble form, this approach eliminates many of the issues such as misfolding of recombinant proteins or separation of nanoparticles and cargo proteins in the target cells. Second, EXPLORs can be readily and easily produced once the appropriate EXPLOR-producing donor cells have been selected from genetically engineered cells. By simply irradiating with a blue light, we can generate EXPLORs loaded with specific cargo proteins, including those derived from other cell types. This stable production of EXPLORs from genetically engineered cells has great potential for the commercialization of standardized exosome-based protein therapeutics. Finally, EXPLORs are compatible with many personalized medicine-based therapeutic approaches. By modifying patient-derived cells to produce EXPLORs^28^, we would be able to manufacture patient-customized drug delivery systems with reduced risk of triggering host immune responses, opening a new paradigm for personalized protein-based therapeutics.

In the present study, we showed that exosomes from cells transiently transfected with a *luciferase-mCherry-CRY2* vector rarely had luciferase molecules; however, EXPLORs that were derived from cells transfected with *luciferase-mCherry-CRY2* and *CIBN-EGFP-CD9* vectors under the light OFF condition had considerable numbers of luciferase molecules. The difference between the two conditions was CIBN-EGFP-CD9 expression. Recently, one research group showed that overexpressed CRY2-conjugated proteins have a weak binding activity with CIBN-conjugated proteins in the normal state^29^. Thus, we suggest that unintended binding of mCherry-CRY2 to CIBN-EGFP-CD9 could be partially responsible for loading of mCherry proteins into exosomes under the light OFF condition.

EXPLORs had the highest efficiency in protein encapsulation under blue light illumination in the power range of 20–50 μW cm^−2^, not at the strongest power (200 μW cm^−2^). We found that incubation of cells under blue light illumination at a power of 200 μW cm^−2^ did not induce significant cell death; thus, cell death was not the cause of decreased protein loading efficiency according to strong light. We presumed that aggregation of CRY2 proteins by strong blue light might disturb the light-induced PPI between mCherry-CRY2 and CIBN-EGFP-CD9 proteins^16^. To address this, we used CRY2 fusion proteins conjugated with Cre recombinase. Unlike other CRY2 fusion proteins conjugated with cytosolic proteins, Cre-mCherry-CRY2 proteins were expressed mainly in the nuclei. Because aggregated forms of Cre-CRY2 proteins cannot diffuse readily through nuclear membranes, we can readily study the effect of light intensity on the aggregation of CRY2 proteins by simply analyzing the subcellular localization of Cre-mCherry-CRY2 proteins. As expected, we observed that the stronger the blue light illuminated, the more Cre-mCherry-CRY2 stayed in the cytosolic compartment, rather than inside the nucleus, confirming that CRY2-fusion proteins can form aggregates under high-intensity light conditions (Supplementary Fig. 20).

We found that one or two cargo molecules were contained in one particle of EXPLOR on average; however, this limited loading efficiency might still have room for improvement. In the present study, we used fusion proteins of fluorescence reporters with both the CIBN and CRY2 protein for better tracking of target proteins. Because we have confirmed that target proteins conjugated with CRY2 can be successfully recruited to engineered exosomes, direct conjugation of cargo proteins or tetraspanins to CRY2 or CIBN proteins without fluorescent reporter proteins can further decrease the molecular size of cargo proteins and subsequently improve loading efficiency of cargo proteins into EXPLORs. In addition, the current strategy using two vectors can further be optimized by using a one-vector system, by introducing an internal ribosome entry site. These approaches might increase the transfection efficiency, as well as the loading of cargo proteins into EXPLORs.

In the present study, we developed a novel protein carrier EXPLOR that has a higher loading capacity and delivery efficiency than previous methods for protein-loaded exosomes. Also, we demonstrated the intracellular delivery of mCherry, Bax, super-repressor IκB protein and Cre enzyme as functional proteins into the target cells *in vitro* and into brain parenchymal cells *in vivo*. Many intracellular proteins, such as transcription factors, signal transducers and enzymes can also be attractive targets for EXPLOR-based therapeutics. Using our EXPLOR technique, we might be able to design various EXPLOR-producing cells that would be equivalent to monoclonal antibody-producing hybridoma cells for protein-based therapeutics targeting intracellular events.

## Methods

### Cell culture

HEK293T human embryonic kidney cells (CRL-3216, ATCC, Manassas, VA, USA), HT1080 cells (CCL-121, ATCC) and HeLa cells (CCL-2, ATCC) were maintained in Dulbecco's modified Eagle's medium (DMEM; Welgene, Seoul, Korea) containing 10% fetal bovine serum (FBS; Gibco, Gaithersburg, MD, USA) and 1% penicillin–streptomycin (Gibco).

### Blue LED-installed incubator

The 460-nm LED bars for plant growth (TG-TECH, Asan, Korea) were installed in a CO_2_ incubator for illuminating cells with blue light. For controlling the light power of the blue LED, a LED dimmer (Hyeonju LED, Seoul, Korea) was added. Automatic control of the on/off cycle was achieved using an infinite-iteration timer attached to the power source (Seojun Electronics, Seoul, Korea). To equally illuminate blue light on the cells, a polycarbonate plate of 3 mm width (BEST ACRYLIC, Incheon, Korea) was added under the blue LED bars as a light diffuser. An optical power meter 8230E (ADCMT, Tokyo, Japan) was used to assess the LED illumination power.

### Reagents

Primary antibody sources were as follows: mouse monoclonal mCherry antibody (diluted 1:1,000 for western blotting, 1:50 for EM, ab125096), rabbit monoclonal CD9 antibody (diluted 1:50 for EM, ab92726) and NF-κB antibodies (diluted 1:1,000 for immunocytochemistry, ab16502) were obtained from Abcam (Cambridge, MA, USA); CD63 (diluted 1:1,000 for western blotting, sc-15363) and GAPDH (diluted 1:4,000 for western blotting, sc-25778) were purchased from Santa Cruz Biotechnology (Santa Cruz, CA, USA); cytochrome c antibody (diluted 1:1,000 for immunocytochemistry, 556432) was from BD biosciences (San Jose, CA, USA); and the GFP antibody (diluted 1:1,000 for western blotting, 2555) was from Cell Signaling Technology (Beverly, MA, USA). Secondary antibody sources were as follows: anti-rabbit (sc-2004) and mouse secondary antibody (sc-2005) were obtained from Santa Cruz Biotechnology, and Alexa Fluor 647 goat anti-mouse IgG (H+L) antibody (diluted 1:1,000 for ICC, A-21235) and Alexa Fluor 488 goat anti-rabbit IgG (H+L) antibody (diluted 1:1,000 for ICC, A-11008) were purchased from Thermo Fisher Scientific (Wilmington, DE, USA).

### Plasmids and transfection

All plasmids and PCR primers used in this study are listed in Supplementary Table 2. *pCMV-CIBN-EGFP* and *pCMV-mCherry-CRY2* (codon-optimized for expression in mammalian cells) were generated^30^. To generate the *pCMV-CIBN-EGFP-CD9* construct, a sequence encoding full-length *CIBN-EGFP* was PCR-amplified from *CIBN-EGFP* constructs, and a gene encoding *CD9* was PCR-amplified from the LN215 cDNA library using gene-specific primers. For the *CIBN-EGFP-CD9* construct, the first fragment (*CIBN-EGFP*-) contained an EcoRI site, a sequence encoding the full-length *CIBN-EGFP*, and a sequence encoding a flexible linker ( SGGGGSGGGGSGGGGS ). The second PCR fragment contained a flexible linker, a sequence encoding full-length *CD9*, and a XhoI site, followed by 15 base pairs of homology to the pcDNA3.1(+) (Invitrogen, Carlsbad, CA, USA) vector. To construct exosomal membrane-targeting sequence-tagged mCherry, the *mCherry*-encoding gene was amplified from an mCherry-CRY2 vector and ligated into an EcoRI site of XPack CMV-XP-MCS-EF1-Puro Cloning Lentivector (System Biosciences, Mountain View, CA, USA). To assemble *pCMV-Cre-mCherry-CRY2*, genes encoding *Cre* were PCR-amplified from the *pCMV-Cre* vector (Addgene plasmid 11916) using gene-specific primers. For the construct, the first fragment (*Cre*-) contained an EcoRI site, a sequence encoding full-length *Cre* except a stop codon and a sequence encoding a flexible linker (SAGGPPVAT). The second PCR fragment (*mCherry-CRY2*) contained the flexible linker, a sequence encoding full-length *mCherry-CRY2* and a XhoI site, followed by 15 base pairs with homology to the pcDNA3.1(+) vector. A *pCAG-loxP-STOP-loxP-ZsGreen* plasmid was obtained from Addgene (plasmid 51269). The *Bax* gene was amplified from the LN215 cDNA library, super-repressor IκB was obtained from Addgene (plasmid 15294) and firefly luciferase gene was obtained from the *pGL4.11[luc2P]* vector (Promega, Madison, WI, USA). Cells were transfected with Effectene Transfection Reagent (Qiagen, Valencia, CA, USA) at a density of 50–80% confluence, according to the manufacturer's protocol.

### Microscopy for cell imaging

Live cells were imaged using a Zeiss LSM 510 confocal microscope with a × 63 Apochromat 1.0 numerical aperture (NA) objective (Carl Zeiss, Thornwood, NY, USA). Laser light (488 nm) was used to induce dimerization between CRY2PHR and CIBN and to image GFP fusion proteins; a 594- nm laser was used to image mCherry-fusion proteins. *Z*-stack and time-lapse imaging were also performed using the confocal microscope, and the three-dimensional reconstruction and real-time images were processed using ImageJ software. An environmental chamber (Harvard Apparatus, South Natick, MA, USA) enclosing the microscope stand was used to maintain the temperature at 37 °C with 5% CO_2_ during live cell imaging. For fluorescence microscope imaging, cells were fixed with 4% paraformaldehyde at 30 min and imaged using a Zeiss Axiovert 200M fluorescence imaging microscope with × 10 Plan-NeoFluar 0.3 NA objective and a 20 × Plan-NeoFluar 0.5 NA objective (Carl Zeiss). The quantification of cell fluorescence was analysed using the Cellprofiler software (Broad Institute, Cambridge, MA, USA).

### Exosome isolation

Cells were collected and transferred to fresh FBS-free DMEM (Welgene) containing 1% penicillin–streptomycin (Gibco) or DMEM containing 1% penicillin–streptomycin and 10% exosome-depleted FBS^31^. After 48 h, medium was collected and centrifuged at 3,000*g* for 15 min to remove cellular debris. Exosome isolation was performed by precipitation or ultracentrifugation. For precipitation, the supernatant was transferred to a sterile vessel and the appropriate volume of ExoQuick-TC Exosome Precipitation Solution (System Biosciences) was added. Samples were mixed by inverting or flicking the tube and then refrigerated overnight. After 24 h, the ExoQuick-TC mixture was centrifuged (1,500*g*, 30 min). After centrifugation, the supernatant was removed and the mixture was spun down again (1,500*g*, 5 min). All traces of fluid were removed by aspiration, and the exosome pellet was resuspended in 100 μl PBS by passaging 5–10 times through a sterile 27-gauge needle (BD Biosciences, San Jose, CA, USA). Then, the suspended exosomes were filtered through a syringe filter (0.2 μm; Sartorius, Goettingen, Germany) to prevent aggregation of exosomes and to remove Exoquick polymers. For ultracentrifugation-based purification of exosomes, supernatants were transferred to an appropriate vessel for the MF-600 ultracentrifuge (Hanil Science Industrial, Incheon, Korea) and centrifuged (120,000*g*, 2 h). After centrifugation, all traces of fluid were removed by aspiration, and the exosome pellet was resuspended in 100 μl PBS by passaging 5–10 times through a sterile 27-gauge needle (BD Biosciences). The suspended exosomes were then filtered through a syringe filter (0.2 μm, Sartorius). The concentration of suspended exosomes was measured using a Nanodrop ND-1000 spectophotometer (Thermo Scientific, Wilmington, DE, USA). The size of the suspended exosomes was measured with a Zetasizer ZS90 dynamic light scattering machine, operating in intensity mode using a 640-nm HeNe laser (Malvern Instruments, Worcestershire, UK). The number of exosome particles was measured by nanoparticle-tracking analysis with a Nanosight NS300 (Malvern Instruments).

### Immunoblotting

Collected cells or isolated exosomes were washed in ice-cold PBS, and suspended in adjusted volumes of buffer, provided with the M-Per mammalian protein extraction kit (Pierce, Rockford, IL, USA). The cells were incubated on ice for 30 min, and the lysates were then cleared by centrifugation at 10,000*g* for 10 min. Lysates were then separated by 12% SDS–polyacrylamide gel electrophoresis, transferred to nitrocellulose membranes (Invitrogen, Carlsbad, CA, USA), and incubated with the appropriate antibodies. The immunoblots were imaged using an enhanced chemiluminescence system (Ab Frontier, Seoul, Korea). Full scans of western blots are in Supplementary Fig. 5.

### Luciferase activity assay

Collected cells or isolated exosomes were washed in ice-cold PBS, and suspended in adjusted volumes of buffer provided with Reporter Lysis Buffer (Promega). A total volume of 20 μl of samples was loaded in a black 96-well plate (SPL Life Sciences, Pocheon, Korea), and 100 μl of Luciferase Assay Reagent (Promega)-reconstituted substrate was added to each reaction manually. Measurements were performed using a Berthold LB942 TriStar^2^ multidetection microplate reader (Berthold, Bad Wildbad, Germany). The integration time was set at 1 s for all measurements. To perform a quantitative luciferase assay, QuantiLum Recombinant Luciferase (Promega) was used to obtain a standard curve. On the basis of the standard curve, a fitting curve and the number of luciferase molecules were calculated.

### Measurement of DNA-binding activity of NF-κB

Nuclear extracts (5 μg) were assayed for the binding activity of p65/c-Rel (NF-κB) using the TransAM NF-κB assay kit (ActiveMotif, Carlsbad, CA, USA) according to the manufacturer's manual^32^.

### Culture of differentiated neurosphere-derived cells

The cerebral cortex tissues from E12.5 *Rosa-EGFP* reporter mice (The Jackson Laboratory, Bar Harbor, Maine, USA) were dissected from the brain^33^. Isolated tissues were incubated with Accutase (Innovative Cell Technologies, San Diego, CA, USA) for 5 min at 37 °C. Cells were grown on an ultralow attachment surface and maintained as neurospheres for 4 days in the presence of basic fibroblast growth factor (20 ng ml^−1^; R&D systems, Minneapolis, MN, USA) and epidermal growth factor (20 ng ml^−1^; Invitrogen) in DMEM/F12 (Gibco) media containing 1% N2 (Gibco), 2% B27 (Gibco) supplements, and 1% penicillin–streptomycin (Gibco). For differentiation, neurospheres were dissociated and cultured on 18-mm cover glass coated with poly-D-lysine (100 mg ml^−1^) and laminin (5 mg ml^−1^) with removal of epidermal growth factor/basic fibroblast growth factor. On the seventh day of differentiation, purified exosomes were treated to the cells and maintained the culture for an additional 3 days. For immunocytochemistry, cells were then fixed with 4% paraformaldehyde and immunostained with anti-β-Tubulin III (Tuj1) (diluted 1:1,000, Sigma-Aldrich, St. Louis, MO, USA, T2200), anti-GFP (diluted 1:1,000, Abcam, ab290) and counterstained with Hoechst 33342 (Invitrogen). Images were acquired on an EVOS FL cell imaging system (Invitrogen) with a EVOS AMEP 4624 (Plan Fluor × 20/0.45) objective lens.

### Animals

Animal care and handling were performed according to the guidelines of the Animal Care and Use Committee of the Korea Advanced Institute of Science and Technology (KAIST, Daejeon, Korea). *Loxp-stop-loxp-eNpHR3.0-EYFP* transgenic mice (The Jackson Laboratory) were generated by mating heterozygous transgenic and wild-type mice (Males, 22 weeks, C57BL/6J background) in a specific-pathogen-free environment. All mice were maintained on a 12/12-h light/dark cycle (light cycle beginning at 6:00) at a temperature of 23 °C. Food and water were supplied *ad libitum*.

### Stereotactic injection of EXPLORs in mouse brain

All mice were anaesthetized with avertin (20 mg ml^−1^ of tribromoethanol, 20 μl g^−1^ intraperitoneal, Sigma-Aldrich, Milwaukee, WI, USA) and placed in a stereotaxic apparatus (David Kopf Instruments, Tujunga, CA, USA). All injections were delivered at a rate of 0.3–0.8 μl min^−1^ via a 10-μl Hamilton syringe (Hamilton Co., Reno, NV, USA) and an injection needle (33-gauge NanoFil Needle Assortment, blunt; WPI, Worcester, MA USA) using a syringe pump (KD Scientific, Holliston, MA, USA). For expression of eNpHR3.0-EYFP, control EXPLORs and Cre:EXPLORs were prepared. In total, 500 μg of EXPLORs were injected into the ventrolateral part of the brain in each mouse (−2.8 mm anteroposterior, −2.2 mediolateral and 3.8 dorsoventral). At 5 days after transfection, mice were killed for visualization.

### Histology and immunohistochemistry of mouse brain

Mice were deeply anaesthetized with avertin (20 mg ml^−1^ of tribromoethanol, 20 μl g^−1^ intraperitoneal, Sigma-Aldrich) and perfused first with heparin and then with 4% formaldehyde diluted in PBS. Brains were removed and post-fixed overnight at 4 °C. Coronal sections (40-μm thick) were acquired using a vibratome (Leica VT1000S; Leica, Rockleigh, NJ, USA) and collected in PBS. Slices were mounted on glass slides with Vectashield mounting medium containing 4′,6-diamidino-2-phenylindole (DAPI; Vector Labs, Peterborough, UK). Fluorescent images (low magnification) were acquired with an Axio Imager 2 fluorescent microscope (Carl Zeiss). Confocal images were acquired with a LSM780 confocal microscope (Carl Zeiss) equipped with a Plan-Apochromat × 20/0.8 M27 objective. Brain sections were incubated in PBS with 0.5% Triton X-100 (Sigma-Aldrich) for 30 min at room temperature. After washing in PBS for 10 min, sections were incubated in a blocking solution composed of 0.5% bovine serum albumin and 0.25% Triton-X 100 for 1 h at room temperature. For NeuN/GFAP immunostaining, sections were incubated with mouse anti-NeuN primary antibody (MAB377; Millipore) and chicken anti-GFAP primary antibody (AB5541; Millipore), diluted 1:1,000 in PBS containing 0.25% Triton X-100, for 15 h at room temperature. After washing twice in PBS for 5 min each, sections were incubated with fluorescein Alexa 647-conjugated anti-mouse IgG secondary antibody (Jackson ImmunoResearch, West Grove, PA, USA) and Alexa 555-conjugated anti-chicken IgG secondary antibody (Jackson ImmunoResearch), and diluted 1:300 in PBST for 1 h at room temperature. Sections were washed twice in PBS for 5 min each and mounted on glass slides with Vectashield mounting medium containing DAPI (Vector Labs).

### Statistical analyses

Data are presented as means±s.e.m. The significance of the difference between two independent samples was determined using Student's *t*-test. Groups were compared using one-way analysis of variance, with Tukey's *post hoc* test applied to a significant main effect. All data were normally distributed and the variances were similar between the groups being statistically compared. Sample size was based on previous experience with experimental variability, and no statistical method was used to predetermine sample or animal sizes. No samples or animals were excluded from the analysis. All experiments were not randomized. The investigators were not blinded to allocation during experiments or outcome assessment.

### Data availability

All data supporting the findings in this study are included in the article, either in the main figures or in the Supplementary Information files.

## Additional information

**How to cite this article:** Yim, N. *et al*. Exosome engineering for efficient intracellular delivery of soluble proteins using optically reversible protein–protein interaction module. *Nat. Commun.* 7:12277 doi: 10.1038/ncomms12277 (2016).

## Supplementary Material

Supplementary Information Supplementary Figures 1-20, Supplementary Tables 1-2, Supplementary Methods and Supplementary References
