# Supplementary material for: Exosome engineering for efficient intracellular delivery of soluble proteins using optically reversible protein–protein interaction module
Source: Nat Commun. 2016 Jul 22;7:12277. doi: 10.1038/ncomms12277 (PMC4961865; doi:10.1038/ncomms12277)
Supplement: Supplementary Information — Supplementary Figures 1-20, Supplementary Tables 1-2, Supplementary Methods and Supplementary References [file ncomms12277-s1.pdf]

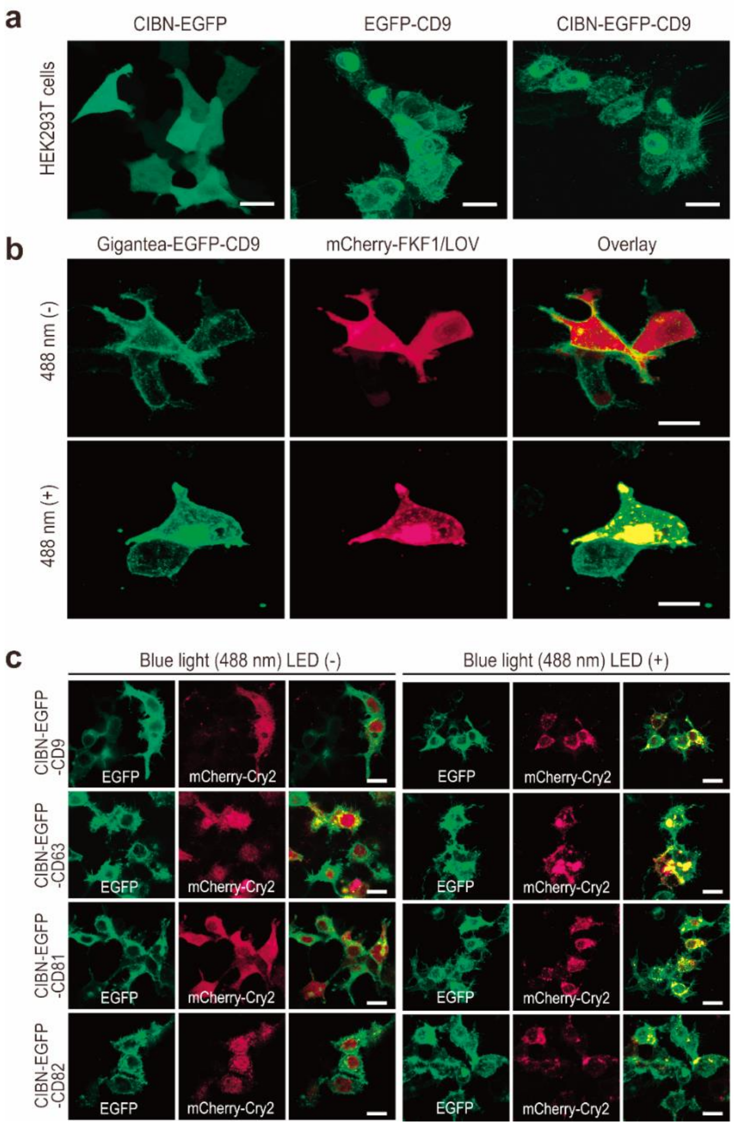

Supplementary Figure 1. The 3D reconstruction images of HEK293T cells transfected with various expression vectors.

(a) The 3D reconstruction images of EGFP fusion protein-expressing HEK293T cells. HEK293T cells were transiently transfected with *CIBN-conjugated EGFP*, *EGFP-tagged CD9*, or *CIBN-conjugated EGFP-tagged CD9*. After 24 h, confocal microscopy revealed the sublocalization of the proteins. Scale bars, 20 μm. A representative result of at least five experiments. **(b** and **c)** 3D reconstruction images of EGFP and mCherry fusion protein-expressing HEK293T cells. HEK293T cells were transiently co-transfected with *Gigantea-EGFP-CD9* and *mCherry-FKF1/LOV* (b), *CIBN-EGFP-CD9*, *CIBN-EGFP-CD63*, *CIBN-EGFP-CD81*, and *CIBN-EGFP-CD82* together with *mCherry-Cry2* (c), respectively. mCherry-CRY2 was imaged both before and after blue light (488 nm) LED exposure. Scale bars, 20 μm. A representative result of at least five independent experiments.

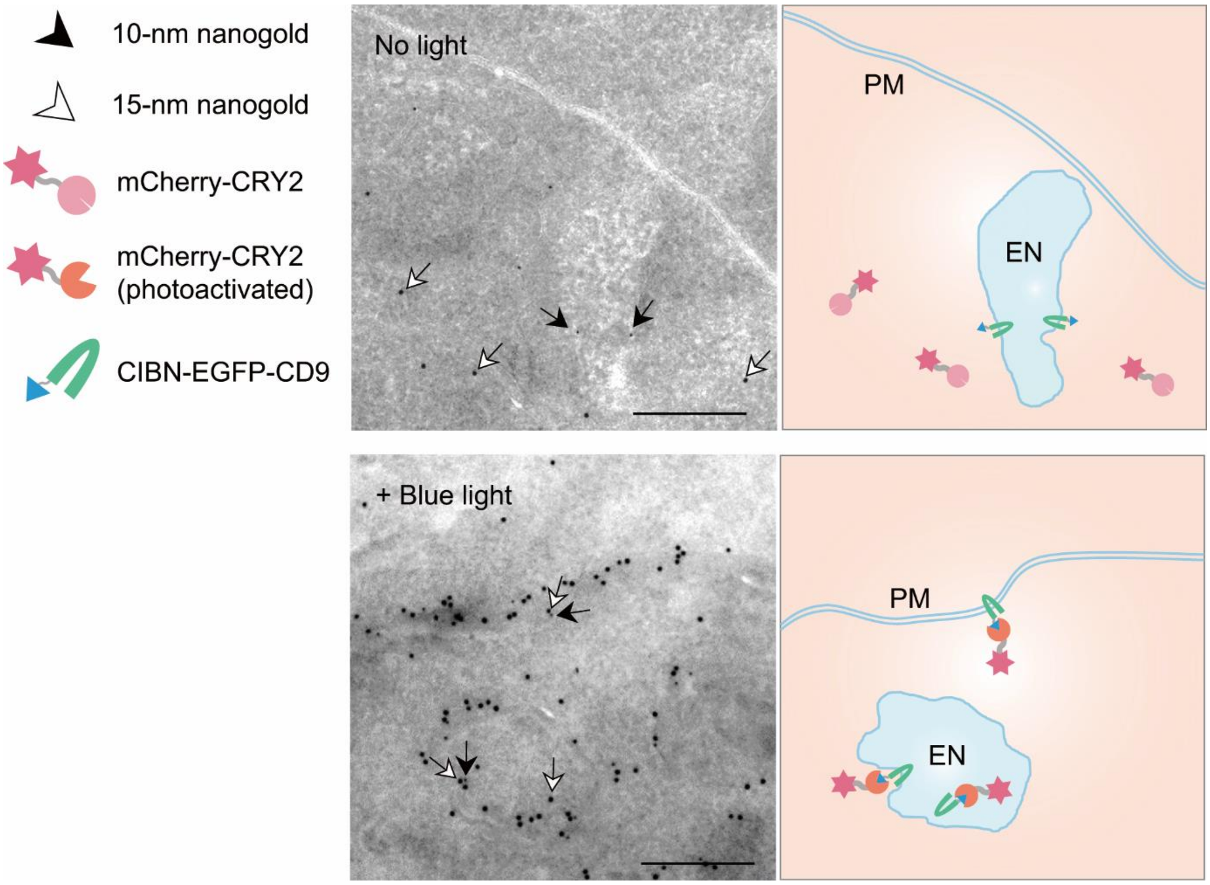

Supplementary Figure 2. Cryo-immunogold electron microscopy analysis of mCherry-CRY2 and CIBN-EGFP-CD9 in HEK293 cells.

Cryo-immunogold electron microscopy images of *mCherry-CRY2* and *CIBN-EGFP-CD9* co-transfected HEK293T cells before and after blue light illumination over 3 h; 15-nm nanogold: mCherry (white filled arrows) and 10 nm nanogold: CD9 (black filled arrows). Schematic images are presented on the right side of each EM image. EN, endosome, PM, plasma membrane. Scale bars, 500 nm. A representative result of at least five independent experiments.

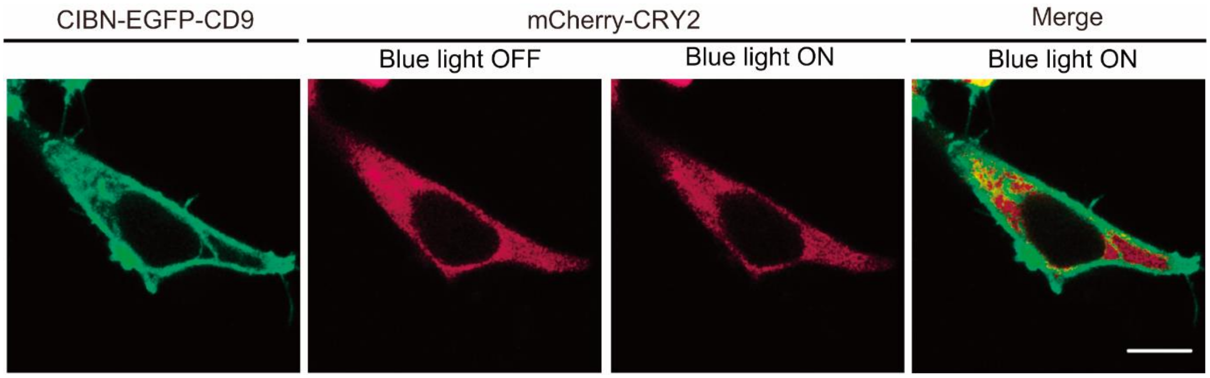

Supplementary Figure 3. Functional validation of the mutant form of mCherry-CRY2PHR (D387A).

HEK293T cells were transiently co-transfected with *CIBN-EGFP-CD9* and *mCherry-CRY2PHR (D387A)*. After 24 h, mCherry-CRY2PHR (D387A) was imaged both before light excitation and after 488-nm laser stimulation (several times; 15 s in duration, 350 μW). Scale bar, 10 μm. A representative result of at least five independent experiments.

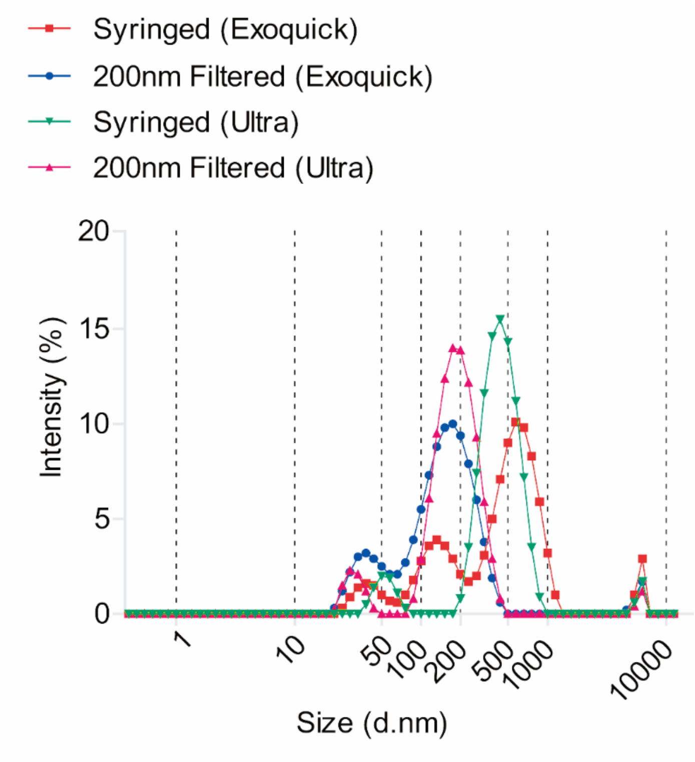

Supplementary Figure 4. Verification of the size of isolated exosomes by dynamic laser scattering (DLS).

HEK293T cell-derived exosomes were extracted by two different methods: precipitation (using Exoquick-TC buffer) and ultracentrifugation. Then, the exosomes were resuspended by syringing through a sterile 27-gauge needle alone, or by both syringing through a sterile 27-gauge needle and filtering through a syringe filter (minisart 0.2 μm). DLS was used to measure the hydrodynamic size and number of the exosomes. The data are summarized in Supplementary Table 2.

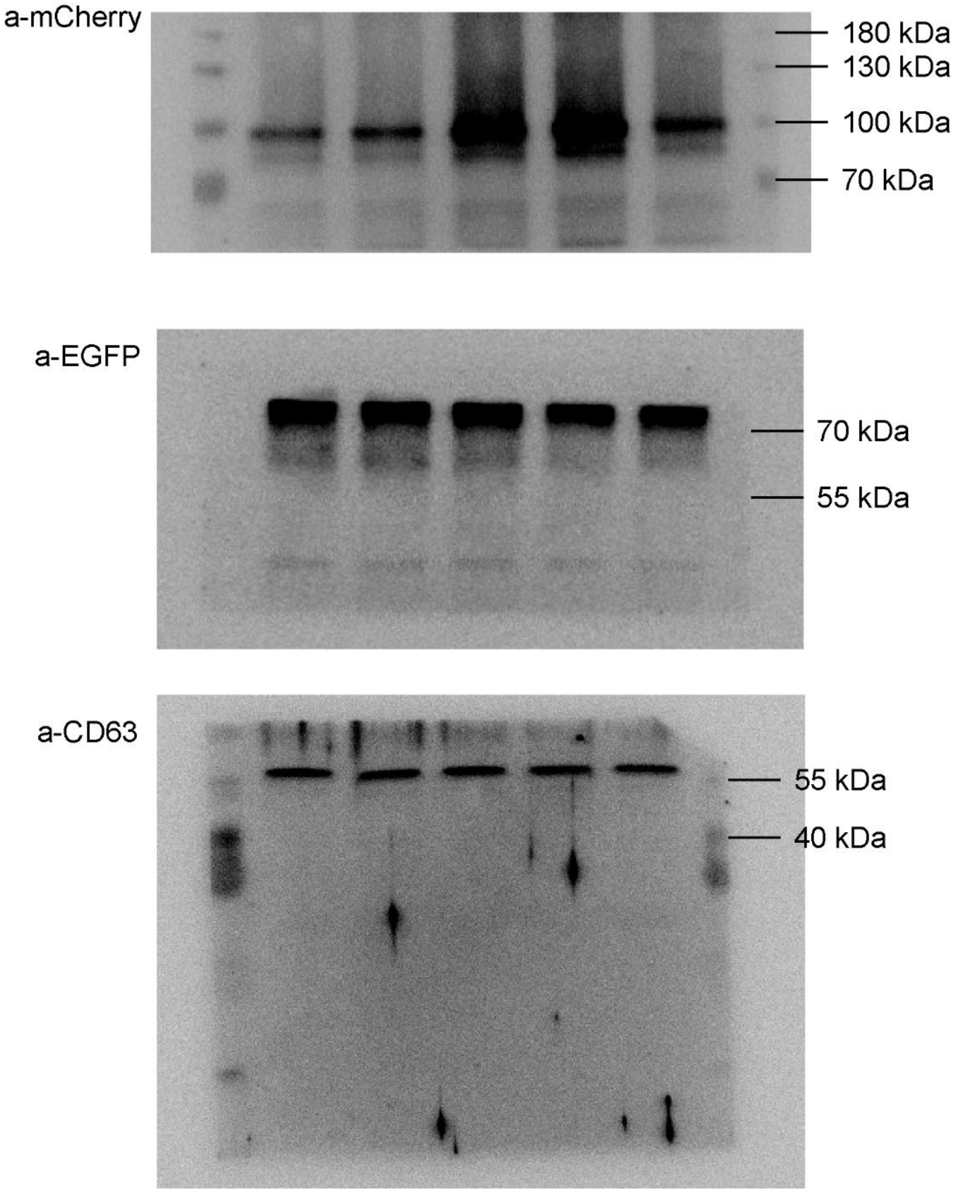

Supplementary Figure 5. Full immunoblots of Main Figure 2.

Cells transiently transfected with CIBN-EGFP-CD9 and mCherry-CRY2 expression vectors were maintained under blue light illumination of varying powers for 48 h. Cell-derived exosomes were subject to immunoblot analysis using antibodies against mCherry-CRY2, CIBN-EGFP-CD9, and CD63, an exosome marker.

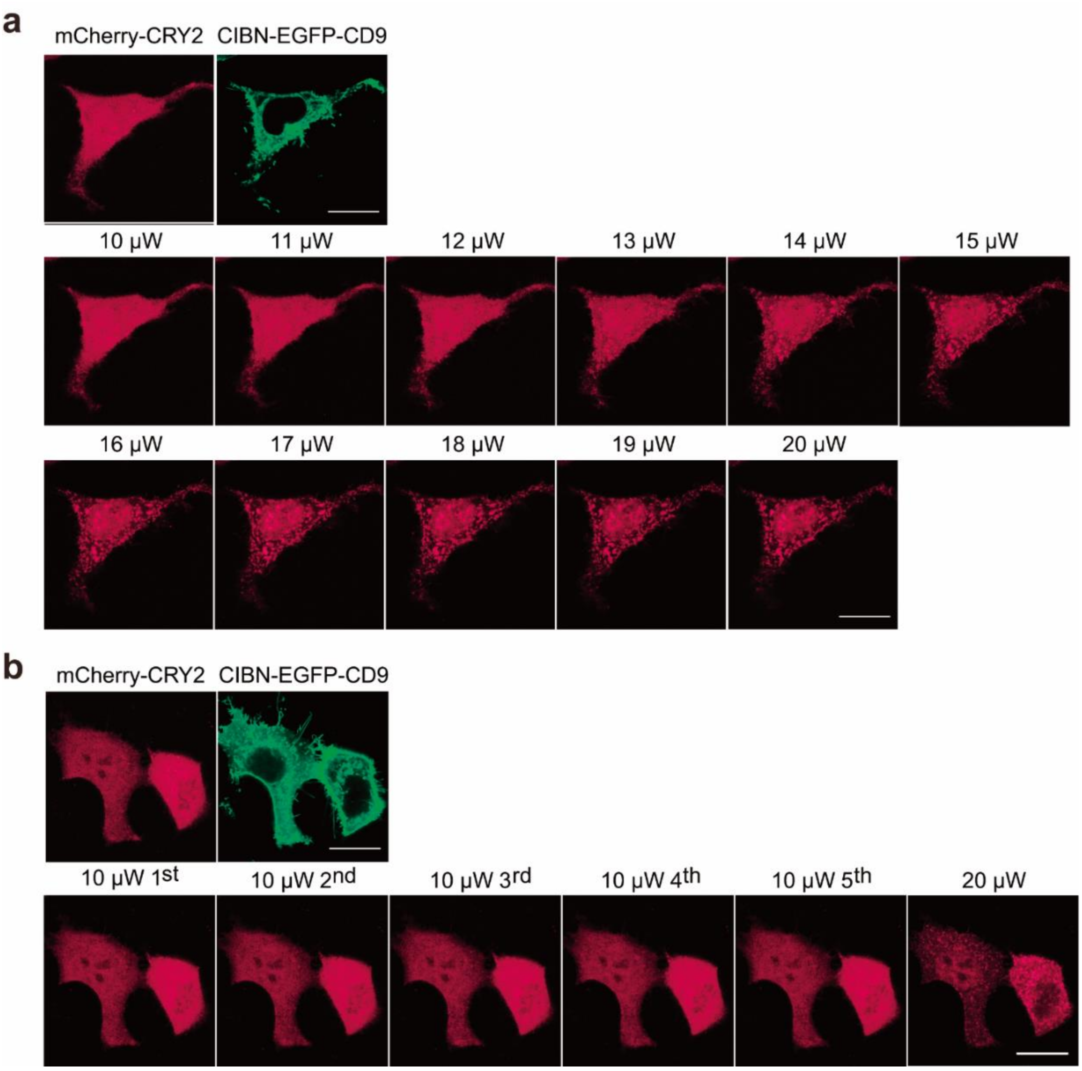

Supplementary Figure 6. Determination of the light power threshold of light-induced protein-protein interaction between CIBN-EGFP-CD9 and mCherry-CRY2 in HEK293T cells.

(a) HEK293T cells were transiently transfected with *mCherry-CRY2* and *CIBN-EGFP-CD9* and incubated for an additional for 24 h. mCherry-CRY2 was imaged by confocal microscopy before light excitation or after various 488-nm laser stimulations (15 s in duration) in the power range of 0-20 µW. Scale bars, 10 µm. A representative result of at least five experiments. **(b)** HEK293T cells were transiently transfected with *mCherry-CRY2* and *CIBN-EGFP-CD9*. After 24 h, mCherry-CRY2 was imaged by confocal microscopy in the following order: before 488-nm laser stimulation, after repeated stimulation at 10 µW (15 s in each duration, total five times), or after stimulation at 20 µW. Scale bars, 20 µm. A representative result of at least five independent experiments.

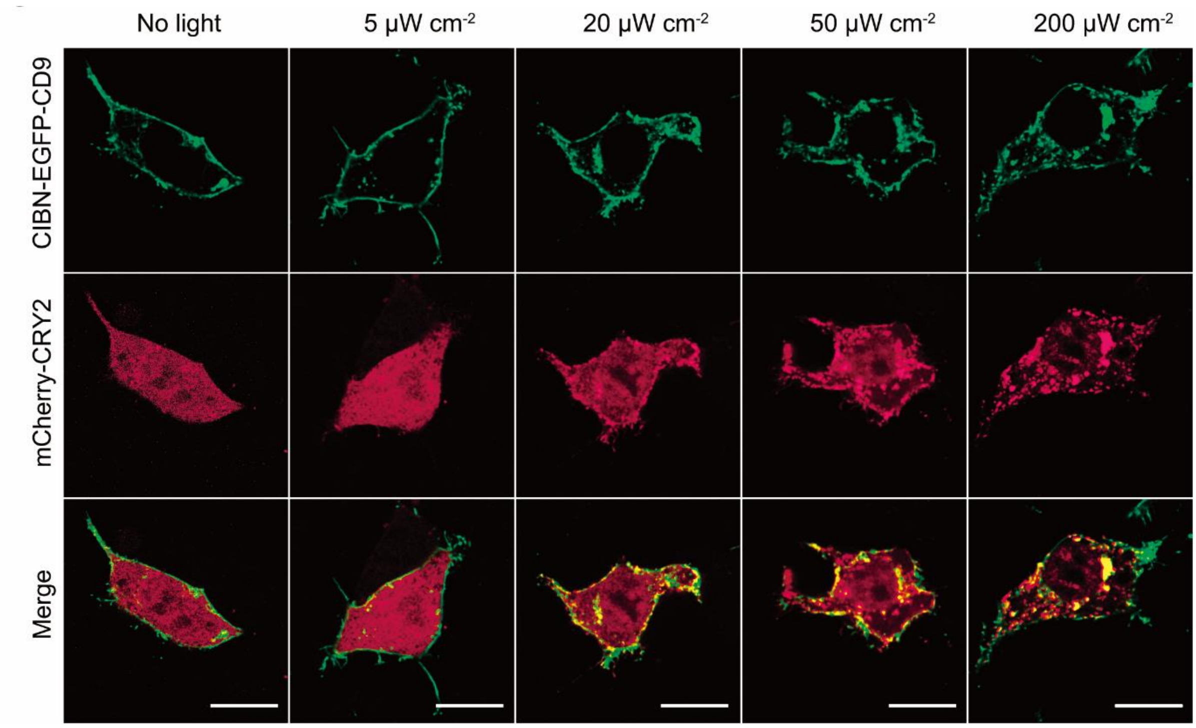

Supplementary Figure 7. Light intensity-dependent protein-protein interaction between CIBN-EGFP-CD9 and mCherry-CRY2 in HEK293T cells.

HEK293T cells transfected with *mCherry-CRY2* and *CIBN-EGFP-CD9* were incubated under no light or blue light in the intensity range of 0-200 μW cm^{-2} for 48 h in the 460-nm LED-installed incubator. After 24 h, cells were fixed with 4% paraformaldehyde, and mCherry and EGFP fluorescence was imaged by confocal microscopy. Scale bars, 10 μm. A representative result from three independent experiments.

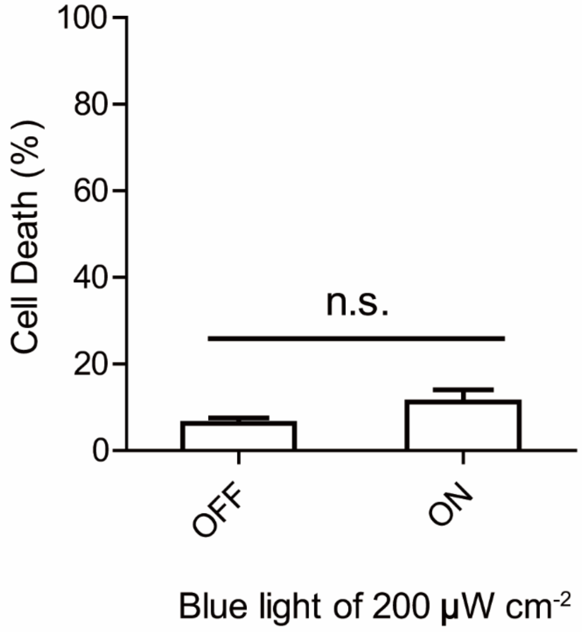

Supplementary Figure 8. Blue light-induced cytotoxicity of EXPLOR-producing cells incubated in blue light ON/OFF conditions.

HEK293T cells were incubated under no light or blue light at a power of 200 μW cm^{-2} for 48 h. Cell death was assessed using an LDH assay. Data are presented as means ± SEM (n = 3) and Tukey's post hoc test was used to test significant group effects identified by ANOVA.

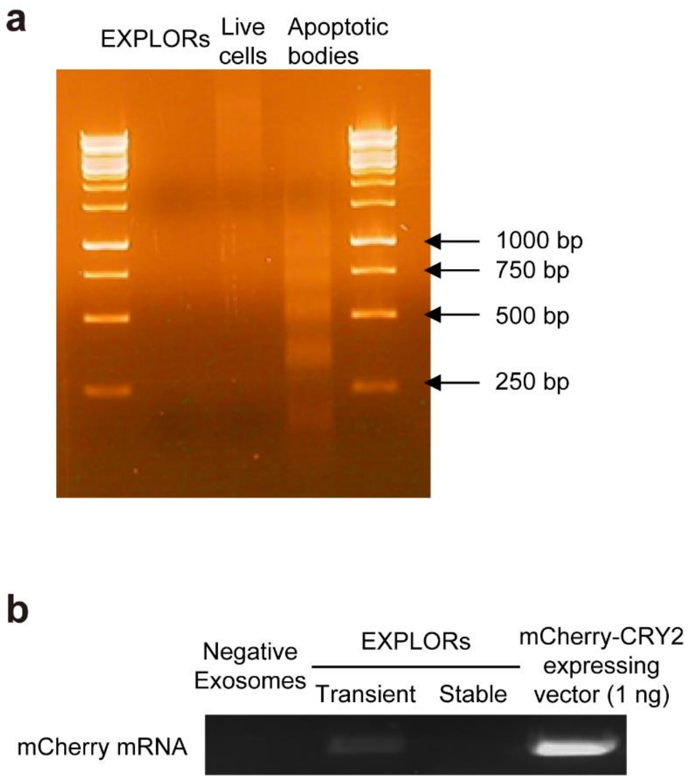

Supplementary Figure 9. Test for DNA fragments and mRNAs in EXPLORs derived from genetically-modified cells.

(a) Comparison between EXPLORs and apoptotic bodies using DNA extraction study. In the EXPLOR group, HEK293T cells were transiently transfected with *mCherry-CRY2* and *CIBN-EGFP-CD9*. After 24 h, cells were incubated under blue light for 48 h in fresh serum-free medium. Then, EXPLORs were isolated according to the Exoquick-TC manufacturer's protocol. In the live cell group, HEK293T cells were maintained normally for 48 h and lysed. In the apoptotic bodies group, cells were incubated and co treated with 1 μM TNF-α and 10 μM MG132 for 24 h. Then, the culture medium was harvested, and apoptotic bodies were collected by centrifugation (2,000×g). DNAs of the three groups were extracted by gene extraction and a purification mini kit (QIAGEN, Hilden, Germany). The GeneRuler 1 kb DNA ladder (ThermoScientific, Rochester, NY, USA) indicates sizes of 250, 500, 750, and 1,000 bp from the bottom band. (b) Test for mRNA in EXPLORs. Negative exosomes derived from HEK293T cells and EXPLORs derived from mCherry-CRY2 and CIBN-EGFP-CD9 transiently or stably co-expressing HEK293T cells were prepared. RNAs were extracted from 1×10^9 particles of the isolated exosomes by a RNeasy Mini kit (QIAGEN), and cDNA library were constructed from the RNAs by a high capacity RNA to cDNA kit (Applied Biosystems, Foster City, CA, USA). A PCR reaction was performed on exosome-derived cDNAs for detecting mCherry mRNA with specific primers, 5'-CACGAGTTCGAGATCGAGGG 3' and 5'-GGTGTAGTCCTCGTTGTGGG 3'; 1 ng of mCherry-CRY2 expressing vector was used for a positive control.

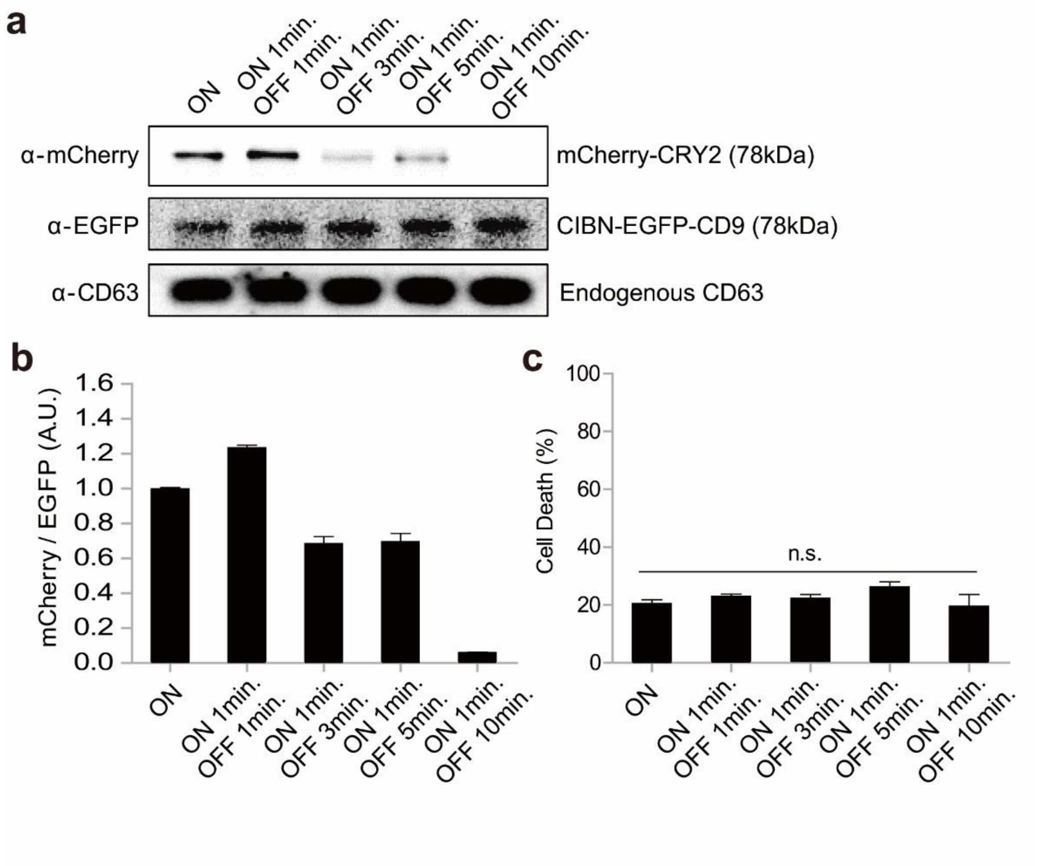

Supplementary Figure 10. Comparison of mCherry packaging efficiency in EXPLORs according to the blue light illumination condition.

(a) mCherry:EXPLORs were isolated from CIBN-EGFP-CD9- and mCherry-CRY2-co-expressing HEK293T cells in various blue light on/off conditions and analyzed by immunoblotting for mCherry and CIBN-EGFP-CD9 proteins. CD63 was used as a loading control. A representative result from three independent experiments. (b) The graph presents the average densitometry values of mCherry-CRY2 proteins per CIBN-EGFP-CD9 proteins in three independent experiments. ON, blue light LED always on; OFF, blue light LED always off; ON 1 min / OFF x min, repeatedly turn on the blue light for 1 min after turning off the light for x min. (c) CIBN-EGFP-CD9- and mCherry-CRY2-co-expressing HEK293T cells were incubated in various blue light on/off conditions for 48 h. Cell death was assessed using an LDH assay. Data are presented as means ± SEM (n = 3), and Tukey's post hoc test was used to test significant group effects identified by ANOVA.

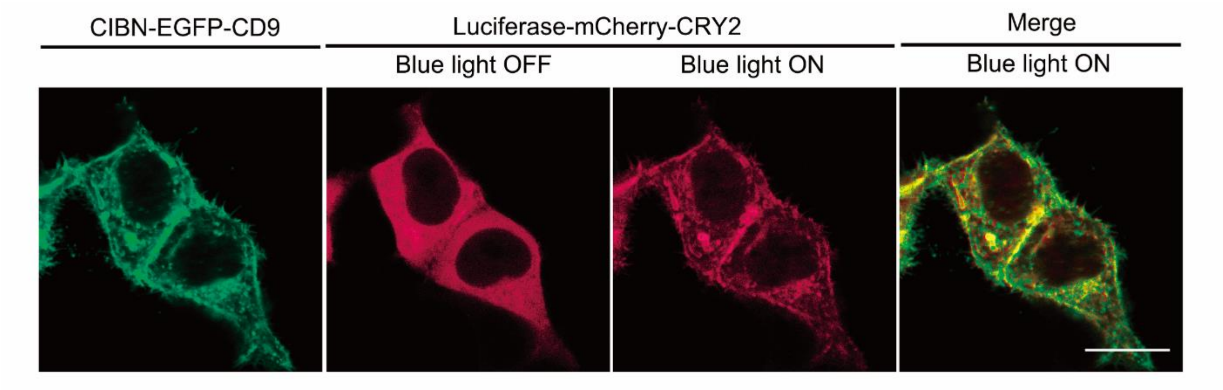

Supplementary Figure 11. Colocalization of luciferase-mCherry-CRY2 with CIBN-EGFP-CD9 in response to light illumination.

HEK293T cells were transiently transfected with CIBN-EGFP-CD9 and Luciferase-mCherry-CRY2 expression vectors. The mCherry fluorescence was imaged before and after 488 nm laser stimulation (15 s in duration, 350 μW cm^{-2}). Scale bars, 20 μm. A representative result of at least five experiments.

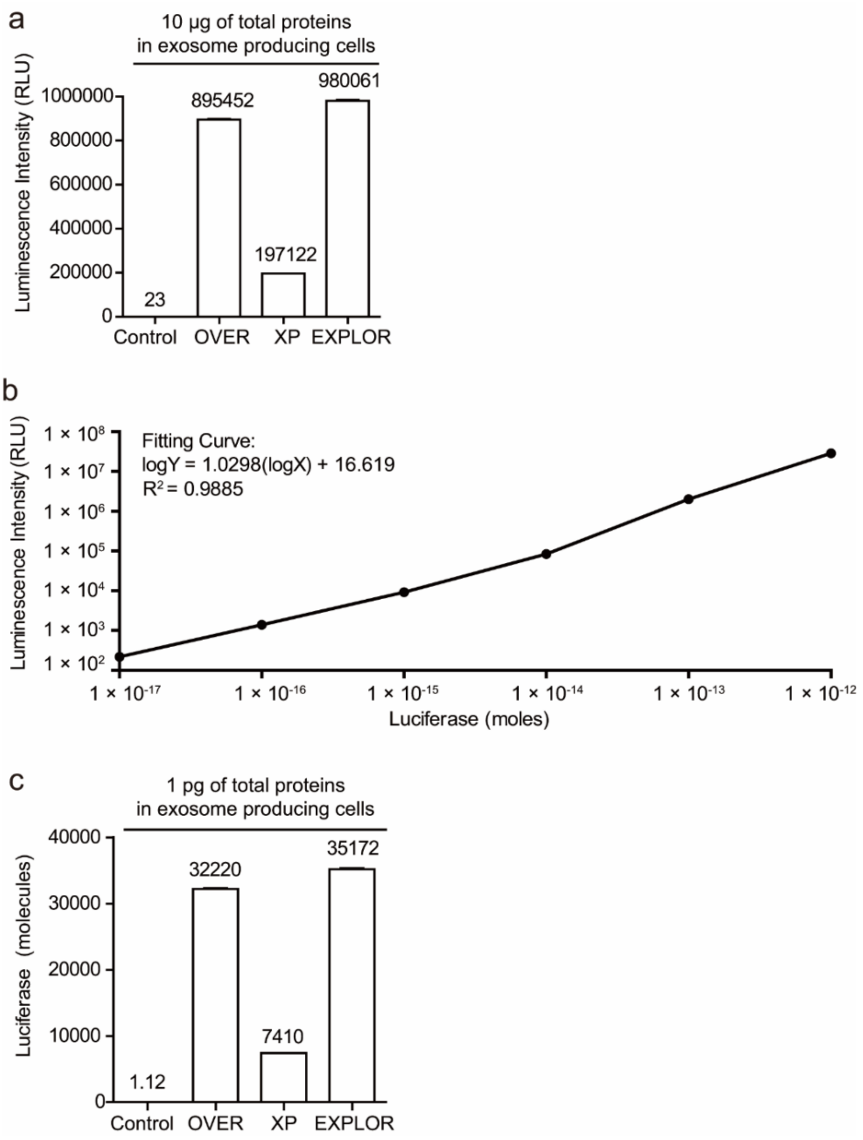

Supplementary Figure 12. Quantitative luciferase assay of exosome producing cells.

(a) HEK293T cells were transiently transfected with mCherry-CRY2 expression vector or XPACK-luciferase-mCherry expression vector, or co-transfected with CIBN-EGFP-CD9 and mCherry-CRY2 expression vectors. After 24 h, cells were lysed and 10 μg of total proteins were analyzed to luciferase activity. Data are presented as means ± SEM (n = 3). (b) A standard curve of recombinant luciferases. (c) The average number of luciferase molecules in 1 pg of total proteins was calculated based on the standard curve of recombinant luciferases. Data are presented as means ± SEM (n = 3). Control, control HEK293T cells; OVER, *luciferase-mCherry-CRY2* transiently transfected cells; XP, *XPACK-luciferase-mCherry* transiently transfected cells; EXPLOR, *luciferase-mCherry-CRY2* and *CIBN-EGFP-CD9* transiently transfected cells.

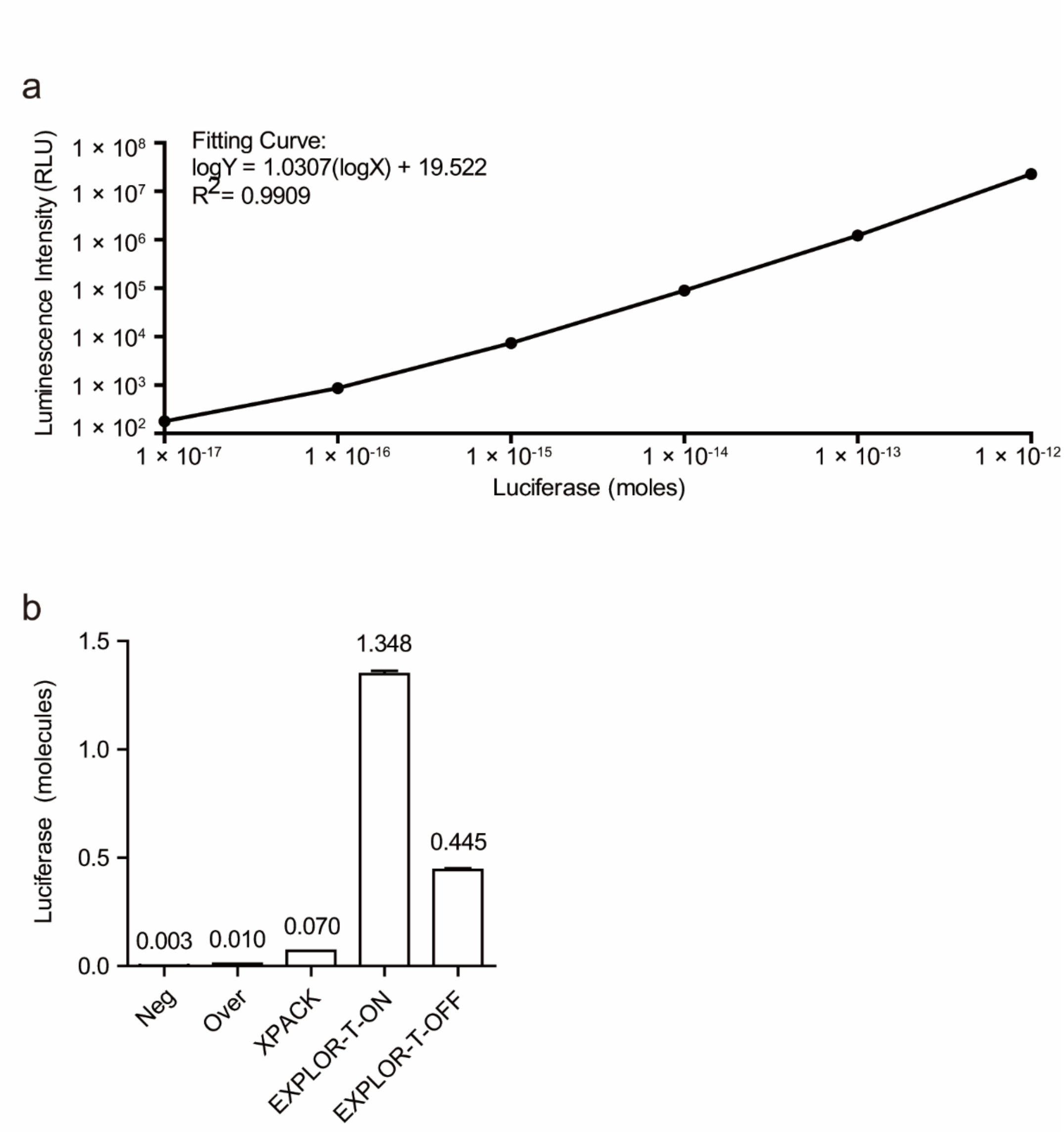

Supplementary Figure 13. Estimation of luciferase molecule number loaed in exosomes.

(a) A standard curve of recombinant luciferases. (b) The average number of luciferase molecules in one exosome were calculated based on the standard curve of recombinant luciferases. Data are presented as means ± SEM (n = 3). NEG, Negative exosomes, OVER, Exosomes from luciferase-mCherry-CRY2 transiently expressing cells, XP, exosomes from XPACK-luciferase-mCherry transiently expressing cells; EXPLOR ON, luciferase-mCherry:EXPLORs produced under the blue light ON condition; EXPLOR OFF, luciferase-mCherry:EXPLORs produced under the light OFF condition.

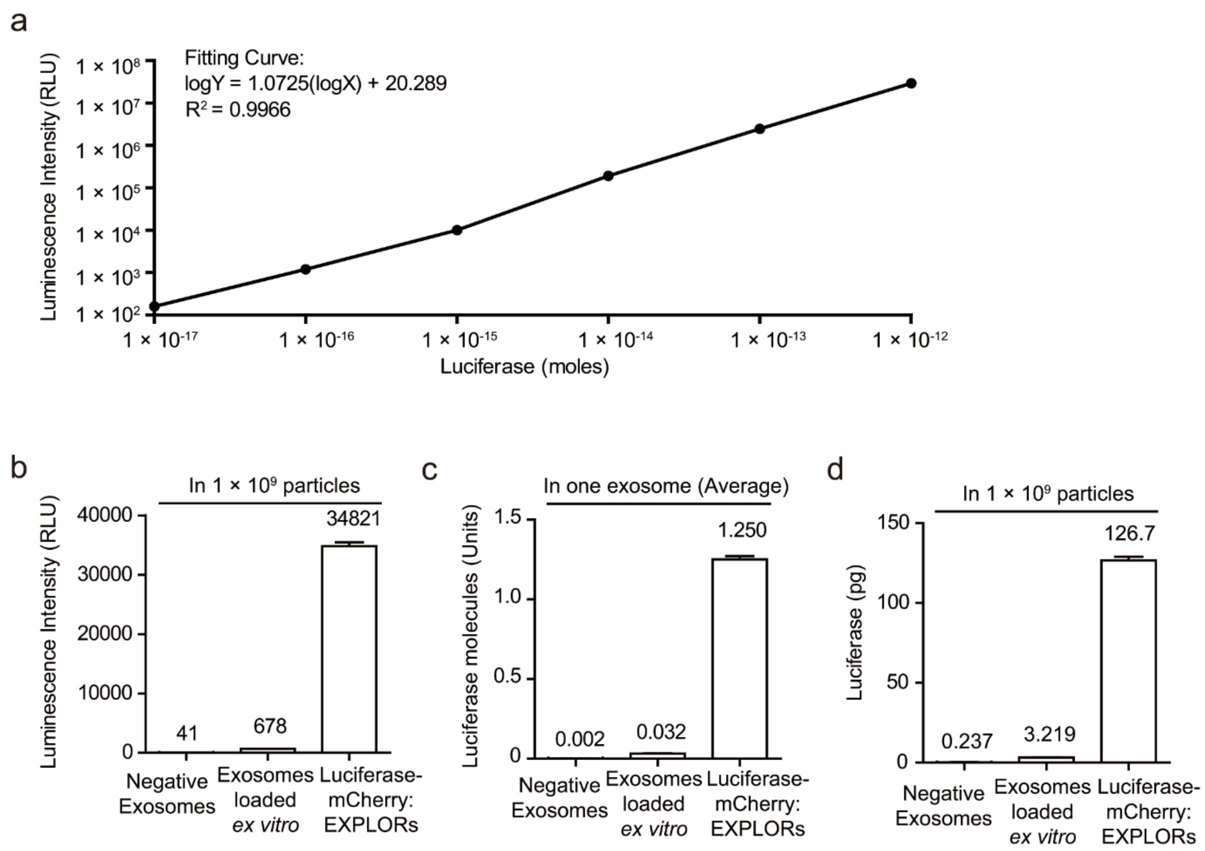

Supplementary Figure 14. Comparison of EXPLOR with ex vitro exosome loading method with recombinant luciferases.

(a) A standard curve of recombinant luciferases. (b) 1×10^9 particles of the isolated exosomes were analyzed for luciferase activity. (c) The average number of luciferase molecules in one exosome was calculated based on the standard curve of recombinant luciferases. (d) The average weights of luciferase molecules in 1×10^9 molecules of the isolated exosomes were calculated. All data are presented as means ± SEM (n = 3).

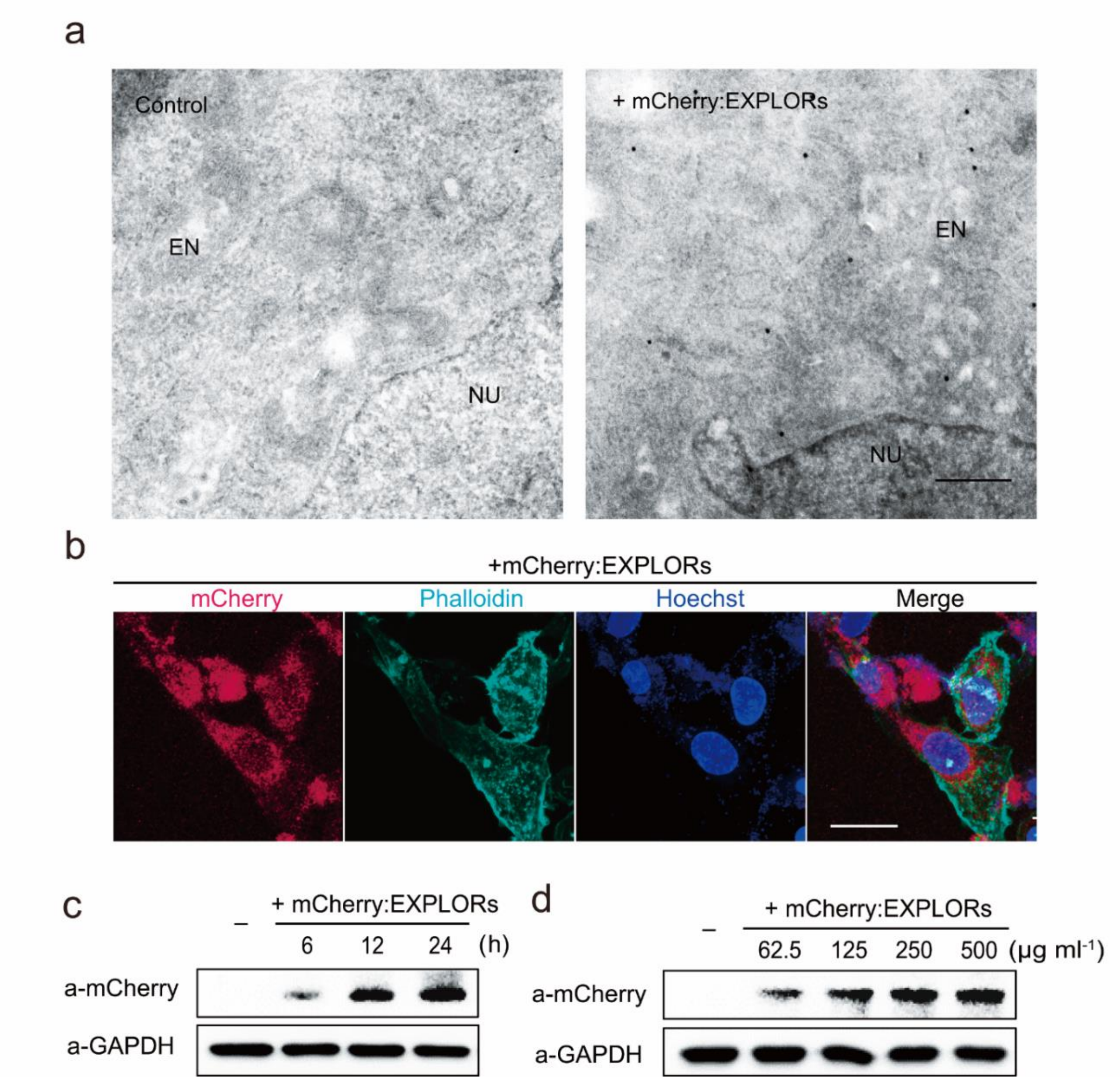

Supplementary Figure 15. EXPLORs-mediated intracellular delivery of mCherry protein.

(a) Immunogold electron microscopy images of EXPLOR-mediated mCherry protein delivery. HT1080 cells were incubated in the absence or presence of 0.25 mg mL^{-1} mCherry:EXPLORs. After 24 h, cells were stained for mCherry with 15-nm nanogold and gold-enhanced labeling (black dots), and imaged by cryo-immunogold electron microscopy. EN, endosome; NU, nucleus. Scale bars, 500 nm. A representative result from three independent experiments. (b) Sublocalization of delivered mCherry proteins was analyzed after staining with Alexa Fluor 488-phalloidin (membrane) in HeLa cells after 24 h treatment with 0.2 mg mL^{-1} mCherry:EXPLORs. Scale bar, 20 µm. A representative result from three independent experiments. (c and d) Cells were incubated in the absence or presence of 0.2 mg mL^{-1} mCherry:EXPLORs for varying time periods or with varying doses of mCherry:EXPLORs for 24 h. Cell lysates were analyzed by immunoblotting for mCherry and GAPDH proteins. A representative result from three independent experiments.

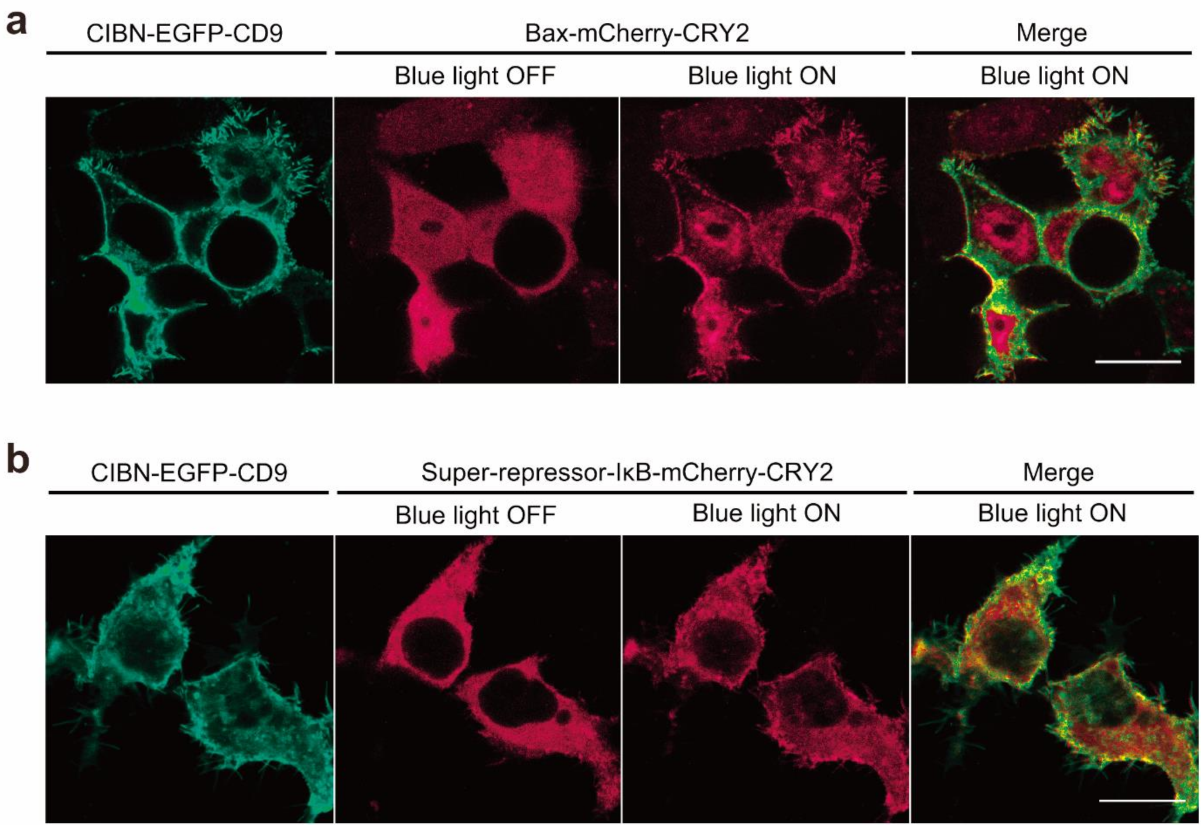

Supplementary Figure 16. Validation of light-induced protein-protein interaction of Bax-mCherry-CRY2 and super-repressor- IκB-mCherry-CRY2 fusion proteins.

(a and b) HEK293T cells were transfected with *CIBN-EGFP-CD9* vectors and *Bax-* (a), or *super-repressor-IκB-mCherry-CRY2* (b). After 24 h of transfection, confocal microscopy revealed the sublocalization of mCherry-fusion proteins and CIBN-EGFP-CD9. The light-induced PPI between mCherry-CRY2-conjugated proteins and CIBN-EGFP-CD9 was examined by 488 nm laser stimulation (15 s in duration, 350 μW cm^{-2}). Scale bars, 20 μm. All data are representative results of at least five experiments.

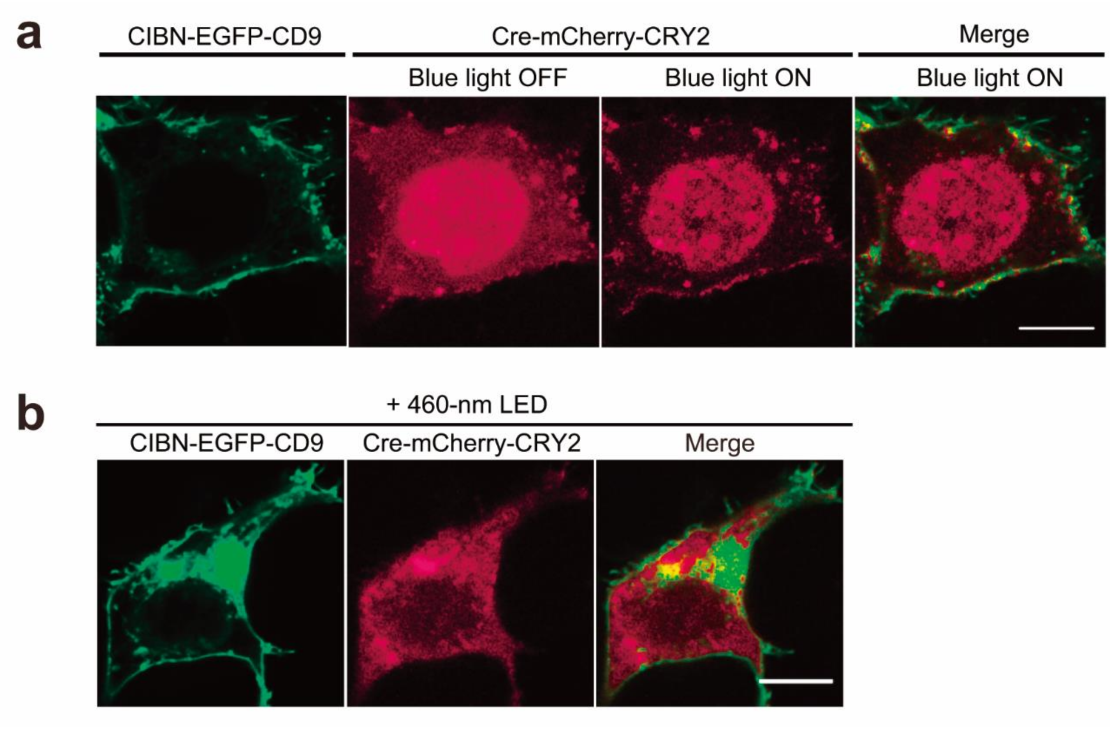

Supplementary Figure 17. Intracellular delivery of Cre-mCherry fusion proteins via EXPLORs.

(a) HEK293T cells were transfected with *CIBN-EGFP-CD9* and *Cre-mCherry-CRY2*. After 24 h of transfection, confocal microscopy revealed the sublocalization of mCherry-fusion protein and CIBN-EGFP-CD9. The light-induced PPI between mCherry-CRY2-conjugated proteins and CIBN-EGFP-CD9 was examined by 488 nm laser stimulation (15 s in duration, 350 μW cm^{-2}). Scale bars, 20 μm. A representative result of at least five experiments. (b) HEK293T cells were transfected with *Cre-mCherry-CRY2* and *CIBN-EGFP-CD9* constructs under blue light illumination in the 460 nm LED-installed incubator. After 24 h, cells were fixed with 4% paraformaldehyde under blue light, and the sublocalization of Cre mCherry-CRY2 and CIBN-EGFP-CD9 was assayed under confocal microscopy. Scale bar, 20 μm. A representative result of at least five experiments.

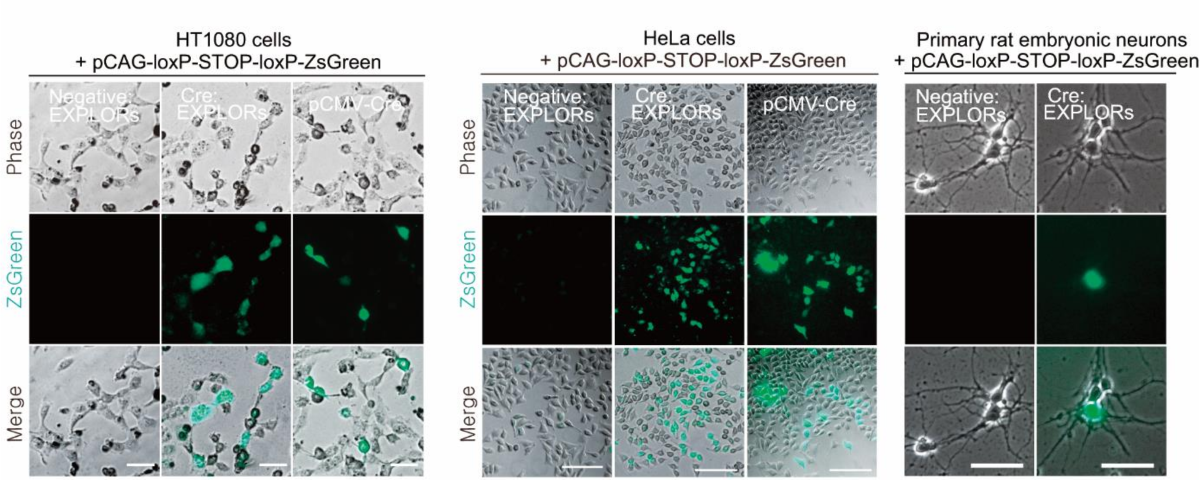

Supplementary Figure 18. Intracellular delivery of functional Cre enzymes via EXPLORs.

HT1080 cells, HeLa cells and primary rat embryonic neurons were transfected with a *pCAG-loxP-STOP-loxP-ZsGreen* reporter construct. At 6 h after transfection, cells were washed and incubated in the absence or presence of 0.25 mg mL^{-1} Cre:EXPLORs or transfected with *pCMV-Cre* vector. After 48 h of incubation with Cre:EXPLORs or transfection with *pCMV-Cre* vector, green fluorescence was measured for ZsGreen reporter protein expression. Scale bars, 40 µm (HT1080), 100 µm (HeLa and primary rat embryonic neurons). A representative result from three independent experiments.

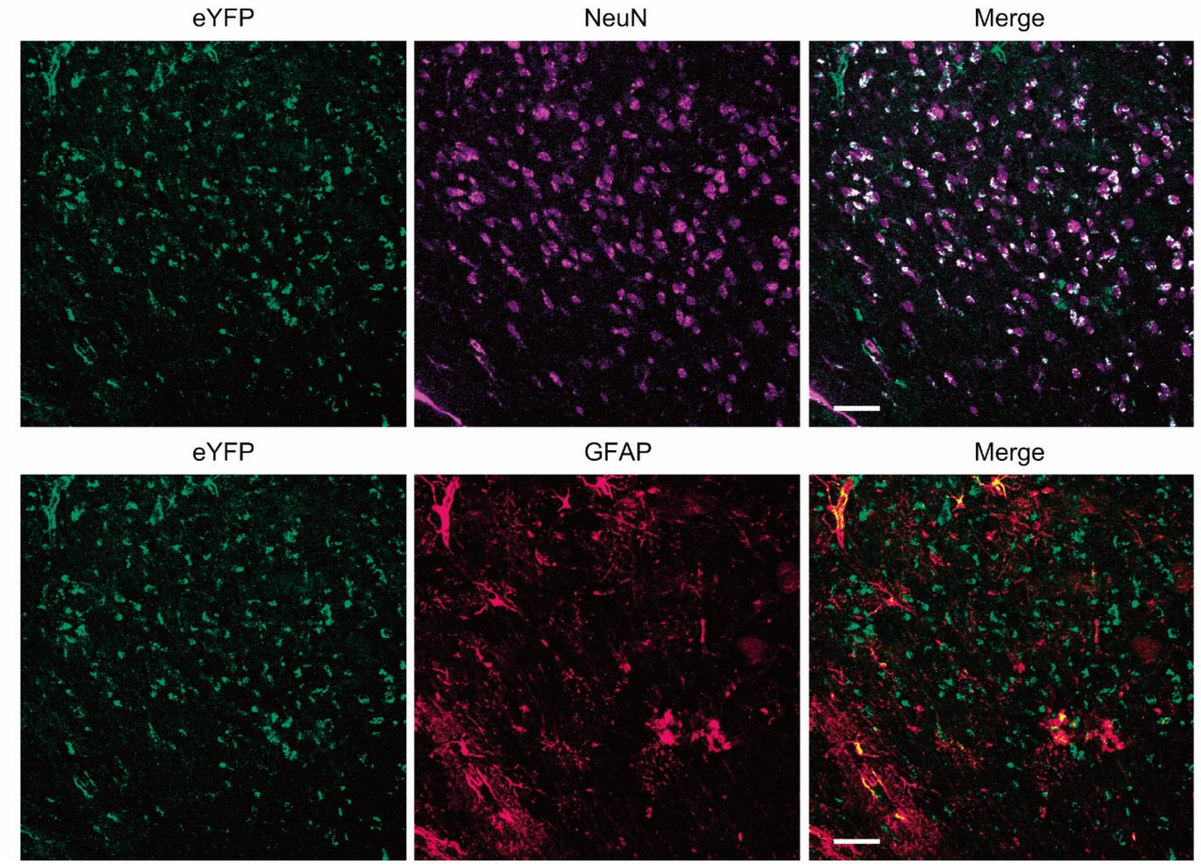

Supplementary Figure 19. Immunohistochemistry analysis of the Cre:EXPLOR-treated brain.

Pink, neuronal specific nuclear protein (NEuN) positive neurons, Red, glial fibrillary acidic protein (GFAP)-positive astrocyte cells. Objective lens, 20×; scale bar, 50 µm.

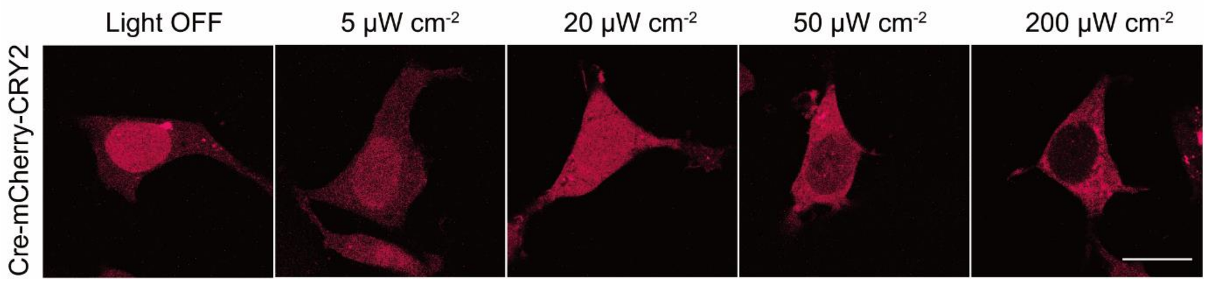

Supplementary Figure 20. Sublocalization of Cre-mCherry-CRY2 according to the power of the blue light in the 460-nm LED-installed incubator.

HEK293T cells transfected with *Cre-mCherry-CRY2* were incubated under light OFF or light ON conditions in the power range from 0 to 200 μW cm^{-2} for 48 h in the 460-nm LED-installed incubator. After 24 h, cells were fixed with 4% paraformaldehyde, and Cre-mCherry-CRY2 were imaged by confocal microscopy. Scale bar, 10 μm.

Exosome Isolation method	Suspension method	Total proteins (mg per 10^7 cells)	Main Peak1	Main Peak2	Main Peak3	Exosomal proteins (mg per 10^7 cells)
			Diameter, nm (Total %)	Diameter, nm (Total %)	Diameter, nm (Total %)	
Exoquick-TC	S*	3.02	555.3 (64.7%)	129.9 (23.5%)	36.41 (7.9%)	0.710
Exoquick-TC	S*+F*	2.46	160.8 (80.2%)	34.39 (17.0%)	5217 (2.8%)	1.97
Ultracentrifuge	S*	0.101	414.9 (90.4%)	46.98 (7.3%)	5370 (2.3%)	< 0.001
Ultracentrifuge	S*+F*	0.065	186.8 (90.8%)	26.36 (7.6%)	5376 (1.6%)	0.059

Supplementary Table 1. Sizes and amounts of total isolated exosomes by dynamic laser scattering (DLS).

The exosomes were isolated by four different isolation methods. * S: syringing with a sterile 27-gauge needle, F: filtering through a syringe filter (minisart 0.2 μm).

DNA fragment for Cloning	Primer sequence	Template
CIBN-Linker	Forward: AGTGTGGTGGAATTCATGAATGGAGCTATAGGAGG<br>Reverse: GCCAGAGCCACCTCCGCCTGAACCGCCTCCACCTGAGAATATAATCCGTTTTCTCC	CIBN-EGFP Vector
Linker-CD9	Forward: GGCGGTTCAGGCGGAGGTGGCTCTGGCGGTGGCGGATCGATGCCGGTCAAAGGAGG<br>Reverse: CCCTCTAGACTCGAGCTAGACCATCTCGCGGTTCC	LN215 cDNA Library
EGFP-Linker	Forward: AGTGTGGTGGAATTCATGGTGAGCAAGGGCGAG<br>Reverse: CCCTCTAGACTCGAGCTAGACCATCTCGCGGTTCC	CIBN-EGFP Vector
Linker-mCherry-CRY2	Forward: AGCGCGGGCGGCCCGCCGGTGGCGACCATGGTGAGCAAGGGCGAG<br>Reverse: CCCTCTAGACTCGAGCTAGGCAGCACCGATCATAATCTGCGC	mCherry-CRY2 Vector
Luciferase-Linker	Forward: TGTGGTGGAATTCTGCAGATATGGAAGATGCCAAAAACATTAAGA<br>Reverse: GCCAGAGCCACCTCCGCCTGAACCGCCACCACCGACGTTGATCCTGGCGCT	pGL4.11[luc2P] Vector (Promega)
Cre-Linker (*Cre-mCherry-CRY2*)	Forward: AGTGTGGTGGAATTCATGTCCAATTTACTGACCGTACACC<br>Reverse: GGTCGCCACCGGCGGGCCGCCCGCGCTATCGCCATCTTCCAGCAGGCGCAC	pCMV-Cre Vector
Cre-Linker (*Cre-CRY2*)	Forward: AGTGTGGTGGAATTCATGTCCAATTTACTGACCGTACACC<br>Reverse: GGTGGCGACCGGTGGACCACCAGCACTATCGCCATCTTCCAGCAGGCGCAC	pCMV-Cre Vector
Linker-CRY2	Forward: AGTGCTGGTGGTCCACCGGTCGCCACCATGAAGATGGACAAAAAGACCATCG<br>Reverse: CCCTCTAGACTCGAGCTAGGCAGCACCGATCATAATCTGCGC	mCherry-CRY2 Vector
Linearized pcDNA3.1(+) (*EcoRI-XhoI Cut*)	Forward: CTCGAGTCTAGAGGGCCCGTTTAA<br>Reverse: GAATTCCACCACACTGGACTAGTG	pcDNA3.1(+)
Bax-Linker (Bax-mCherry-CRY2)	Forward: AGTGTGGTGGAATTCATGGACGGGTCCGGGG<br>Reverse: GCCAGAGCCACCTCCGCCTGAACCGCCTCCACCTGAGCCCATCTTCTTCCAGA	LN215 cDNA Library
Super repressor IκB (super repressor IκB-mCherry-CRY2)	Forward: TGTGGTGGAATTCTGCAGATATGTTCCAGGCGGCCGAG<br>Reverse: GCCAGAGCCACCTCCGCCTGAACCGCCACCACCTAACGTCAGACGCTGGCCT	Addgene Plasmid #15294
-mCherry- (XPACK System)	Forward: ATCCAAGCGGCCGCGAGAATTCGGATGGTGAGCAAGGGCGAG<br>Reverse: CTGCAGATGCTAGCGAGAATTCCTACTCGTCCATGCCGCCGGT	mCherry-CRY2 vector
Luciferase-mCherry (XPACK System)	Forward: ATCCAAGCGGCCGCGAGAATTCGGATGGAAGATGCCAAAAACATTAAGA<br>Reverse: CTGCAGATGCTAGCGAGAATTCCTACTCGTCCATGCCGCCGGT	Luciferase-mCherry-CRY2 vector

Supplementary Table 2. Plasmids and PCR primers.

SUPPLEMENTARY MATERIALS AND METHODS

Cryo-immunogold electron microscopy

Cryo-immunogold electron microscopy was performed according to Kweon et al[1]. Briefly, HEK293T cells were fixed in 4% PFA and 0.01% GA in 0.1 M phosphate buffer, pH 7.4. The fixed cells were then embedded in 10% gelatin. Small gelatin blocks containing cells were infused with 2.3 M sucrose overnight and then frozen in liquid nitrogen. Ultrathin cryosections (45 nm) were cut at -120°C with a cryo-ultramicrotome (UCT7, Leica, Vienna, Austria). Ultrathin sections were obtained using a diamond knife with 2.3 M sucrose: 2% methylcellulose (1:1) and transferred onto Formvar-coated copper grids. All antibodies and gold conjugates were diluted in 0.1% BSA-c (Aurion, Wageningen, the Netherlands) in PBS. The rabbit monoclonal anti-CD9 antibody (Abcam, diluted 1:50) and the mouse monoclonal anti-mCherry antibody (Abcam, diluted 1:50) were used as primary antibodies.

Protein A-gold (from the Department of Cell Biology, Utrecht School of Medicine, Utrecht, the Netherlands) was used to detect primary antibodies. For the double immunogold labeling experiments, the ultrathin cryosections were incubated first with the primary antibody for 30 min after blocking with 0.1% cold fish gelatin and 5% bovine serum albumin for 10 min. The sections were then incubated with protein A-gold for 30 min. Then, a second primary antibody and protein A-gold were applied consecutively. After finishing the antibody labeling step, the grids were then stained with 4% neutral uranyl acetate and embedded in 2% methyl cellulose containing 0.3% uranyl acetate, as described by Tokuyasu[2]. Grids were examined at 120 kV using a Tecnai G^2 Spirit Twin TEM (FEI, Hillsboro, OR, USA) or JEM-1400 Plus (Jeol, Tokyo, Japan).

Detection of cell death

Cells were seeded in 35 mm dishes and washed twice with serum-free DMEM high-glucose medium. After the experiments, the supernatants were analyzed with a lactate dehydrogenase release assay (Promega, Madison, WI, USA) according to the manufacturer’s protocol.

***Ex vitro* protein loading into exosomes**

Naive exosomes were isolated from HEK293T cells and diluted in PBS. 500 µg of QuantiLum® Recombinant Luciferase (Promega) in PBS was added to 2×10^{10} of exosomes. And then, the luciferase mixture with exosomes was extruded (×10 times) through Avanti Lipids extruder (Avanti Polar lipids Inc., Alabaster, AL, USA) with 200 nm-pore diameter[3]. Loaded with luciferase exosomes were purified from free luciferase by gel-filtration chromatography with Sepharose 2 BCL (GE Healthcare, Waukesha, WI, USA) and also by membrane-filtration with Amicon ultra-0.5 centrifugal filter unit with ultracel -100 (Millipore, Bedford, MA, USA).

Primary rat embryonic neuron culture and transfection

All sample preparations were performed in compliance with Institutional Review Board (IRB project number: 2013-ME09) guidelines and regulations. Hippocampi were isolated from E18 Sprague Dawley rats (Orient Bio, Seongnam, Korea) and gently titurated using 0.25% (w/v) trypsin at 37°C for 15 min[4]. Neurons were plated on poly-L-lysine-coated glass coverslips with neurobasal® medium (Gibco) accompanied with B-27, 2 mM Glutamax, and penicillin/streptomycin for 7 - 12 days. The neurons were transfected with pCAG-loxP-STOP-loxP-ZsGreen by electroporation. Briefly, DNA vectors were dissolved in Ca^{2+}-

containing PBS at 40 μg μL^{-1} concentration[5]. The cell suspension was mixed with the solution at room temperature, then transferred to a 1-mm cuvette. The neurons were transiently transfected with a Gene Pulser Xcell Electroporation System (BioRad, Hercules, CA, USA) using a 112-V, 5-ms square pulse. At DIV7, the *pCAG-loxP-STOP-loxP-ZsGreen*-transfected neurons were treated with EXPLOR at a density of 0.16 mg mL^{-1}, and maintained up to 4 days for the experiments.

SUPPLEMENTARY REFERENCES

1. Kweon HS, *et al.* Golgi enzymes are enriched in perforated zones of golgi cisternae but are depleted in COPI vesicles. *Molecular biology of the cell* **15**, 4710-4724 (2004).

2. Tokuyasu KT. Immunochemistry on ultrathin frozen sections. *The Histochemical journal* **12**, 381-403 (1980).

3. Haney MJ, *et al.* Exosomes as drug delivery vehicles for Parkinson's disease therapy. *J Control Release* **207**, 18-30 (2015).

4. Beaudoin GM, 3rd, *et al.* Culturing pyramidal neurons from the early postnatal mouse hippocampus and cortex. *Nat Protoc* **7**, 1741-1754 (2012).

5. Martinez CY, Hollenbeck PJ. Transfection of primary central and peripheral nervous system neurons by electroporation. *Methods Cell Biol* **71**, 339-351 (2003).
